# Self-Perceptions of Aging in Older Adults: A Network Analysis of Clinical and Non-Clinical Samples

**DOI:** 10.3390/brainsci16020204

**Published:** 2026-02-09

**Authors:** Lysiane Le Tirant, Maxim Likhanov, Marie Mazerolle, Alexandrine Morand, Francis Eustache, Pascal Huguet, AGING Consortium, Isabelle Régner

**Affiliations:** 1 Centre de Recherche en Psychologie et Neurosciences, Aix Marseille Université, CNRS, CRPN, 13003 Marseille, France; lysiane.le-tirant@univ-amu.fr (L.L.T.); maksim.likhanov@univ-amu.fr (M.L.); 2 Laboratoire de Recherches Intégratives en Neurosciences et Psychologie Cognitive (LINC), Maison des Sciences de l’Homme et de l’Environnement (MSHE), Université Marie et Louis Pasteur, 25000 Besançon, France; marie.mazerolle@umlp.fr; 3 Laboratoire DysCo, Université Paris 8, 93526 Saint-Denis, France; alexandrine.morand@univ-paris8.fr; 4 Neuropsychologie et Imagerie de la Mémoire Humaine (NIMH), INSERM, U1077, EPHE-PSL, Université de Caen Normandie, 14000 Caen, France; francis.eustache@unicaen.fr; 5 LAboratoire de Psychologie Sociale et Cognitive (LAPSCO), Université Clermont Auvergne, CNRS, 63000 Clermont-Ferrand, France; pascal.huguet@uca.fr

**Keywords:** memory clinic, memory deficits, aging perceptions, negative stereotypes, subjective age, network analysis, cognitive aging

## Abstract

**Highlights:**

**What are the main findings?**

- Subjective age emerged as the most central node for patients, linking all psychosocial variables, whereas Mental deterioration is the one in the representational system in controls.
- Patients showed no cluster of self-perceptions of aging variables, while controls displayed a two-cluster structure separating negative emotional and threat-related variables from the positive views of aging.

**What are the implications of the main findings?**

- The central role of subjective age highlights that patients may rely more on external feedback to evaluate themselves, whereas controls may interpret their aging through socially shared negative stereotypes.
- The absence of clustering in patients may reflect the heterogeneity of their cognitive and pathological profiles, whereas the structured clustering in controls suggests that, outside a clinical context, representations of aging are more organized.

**Abstract:**

**Background:** Cognitive aging is highly heterogeneous, not only in performance but also in how individuals perceive their own aging. Such self-perceptions may shape emotional reactions and adaptation to memory difficulties, yet little is known about their organization in patients referred to a memory clinic for a first diagnostic consultation. The primary aim of this study was to identify the internal configuration of self-perceptions of aging in such patients. A secondary aim was to compare these patterns with those observed in older adults recruited in a research unit of experimental psychology, who reported subjective complaints but had no medical referral. **Methods**: In total, 130 memory clinic patients and 84 laboratory participants completed, prior to the same neuropsychological testing, a psychosocial questionnaire assessing four domains: self-perceptions of memory deficits, attitudes toward aging, aging stereotypes, and multiple facets of subjective age. Network analysis was applied to examine how these variables were interrelated and to determine their relative importance in each group. **Results**: Across both samples, network analyses revealed distinct organizational patterns. Patients showed a unified representational system characterized by more positive associations and centered on subjective age variables. By contrast, the laboratory group showed a two-cluster network with more negative connections, organized around negative aging stereotypes. **Conclusions**: These findings provide novel insights into the psychosocial profile of memory clinic patients, highlighting the added value of network approaches for capturing the interrelations among key self-representations of aging and memory, and for informing and contextualizing clinical evaluation.

## 1. Introduction

The interindividual variability of cognitive aging is well documented on both typical and pathological trajectories. Among cognitively healthy older adults, individuals differ greatly in the level and rate of age-related cognitive changes [1,2,3]. Likewise, early pathological aging—such as in prodromal or preclinical Alzheimer’s disease—also exhibits marked heterogeneity in symptom onset, progression, and cognitive profiles. For example, prior research has identified distinct atrophy subtypes in Alzheimer’s that may account for the disease heterogeneity [4]. Beyond this objective cognitive variability, older adults also differ in how they perceive and interpret their own aging, including the extent to which they emotionally respond to the possibility of pathological decline and perceive the causes, consequences, and controllability of their memory difficulties [5,6,7]. These subjective differences are shaped by several psychosocial factors such as the emotional burden associated with the aging process [8], the internalization of negative aging stereotypes [9,10], and discrepancies in subjective age [11,12,13]. These psychosocial factors are critical components likely to modulate cognitive performances [10] and affect the interpretation and management of symptoms [14]. While the heterogeneity of cognitive aging trajectories is well established, far less is known about the nature and the variability in how psychosocial factors related to aging and memory are organized interconnected.

The clinical relevance of these psychosocial factors becomes especially salient when older adults attend a memory clinic for the first time, a context in which these factors remain surprisingly understudied. In this setting, older adults are likely to face uncertainty, emotional burden, and heightened concerns about the meaning and potential implications of their memory difficulties [15,16]. Importantly, older adults referred to a memory clinic do not constitute a homogenous group: patients differ widely in the severity and chronicity of their complaints, in their worries about possible decline, and in the expectations they bring to the consultation [17,18,19]. In addition, the motivation for attending the clinic may vary substantially, ranging from personal concerns to pressure from family members or recommendation by a general practitioner, which can increase anxiety related to the visit [20]. The first neuropsychological evaluation is thus a critical moment for the patient, where uncertainty and diagnostic anticipation may heighten emotional distress and concerns about cognitive decline. In such a context, psychosocial factors may not only be intensified but may also become organized differently in relation to one another, shaping how memory difficulties are experienced and interpreted.

Among these psychosocial factors, negative aging stereotypes constitute a particularly well-documented source of vulnerability, although this evidence comes exclusively from laboratory studies rather than clinical settings [21]. Experimental research has shown that activating negative age-related stereotypes can impair older adults’ performance on memory tasks [10], as well as on brief cognitive tests similar to those used in memory clinics [22,23,24] and can reinforce the subjective sense of memory decline [25]. These findings underscore the importance of considering how internalized stereotypes may shape older adults’ expectations and reactions when they seek memory assessment.

Likewise, another essential set of psychosocial factors, illness perceptions [26,27], provides a useful framework for understanding older adults’ reactions to cognitive decline. Research grounded in the Common Sense Model of Leventhal et al. has shown that these illness perceptions encompass several distinct dimensions: symptoms identification, timeline (expected duration and severity), perceived causes, consequences, control (personal or treatment-related), emotional representation, and illness coherence [14]. These perceptions are highly subjective and acquired through personal experiences and social environments (e.g., family and friends, media, health professionals). They shape how individuals interpret their symptoms and anticipate their progression, thereby influencing coping mechanisms, help-seeking behaviors, and health-related decision-making [27,28]. This highlights the importance of considering how older adults conceptualize their memory deficits, as these beliefs are likely to guide their responses and engagement in care.

In addition to illness-specific beliefs, older adults’ interpretations of memory difficulties are embedded within broader self-perceptions of aging. Although aging is a natural and universal process, its experience is complex and involves emotional and physical challenges that require ongoing adaptation [29]. In this context, self-perceptions of aging play a crucial role. Specifically, positive views of aging are associated with greater engagement in health-promoting behaviors and coping strategies, whereas negative views are linked to poorer health outcomes, often through denial or avoidance coping strategies [28,30,31]. And because cognitive decline, including memory difficulties, commonly accompanies aging, the experience of memory deficits may trigger fears of future dementia [32]. Collectively, these findings indicate that beyond illness-specific beliefs, broader self-perceptions of aging play a central role in shaping their interpretation of memory problems and making decisions regarding their management.

Finally, subjective age, defined as how individuals perceive their own aging process, also plays a significant role in shaping perceptions of aging [33]. In the literature, subjective age is considered a meaningful indicator of aging trajectories, offering insight into both cognitive functioning and overall health [34]. Evidence further indicates that feeling younger than one’s chronological age helps maintain more positive views of aging [12] and is associated with better physical and mental health outcomes [33,35]. These perceptions, in turn, can influence health-related decision making, encourage engagement in health-promoting behaviors, and buffer against negative emotional responses such as anxiety and stress [30,34]. Overall, subjective age displays a key role in how individuals view their aging process and health, therefore influencing their responses to symptoms and approach to health management.

Despite the acknowledged relevance of psychosocial factors in the experience of cognitive aging, little is known about how these factors are jointly organized in older adults attending a memory clinic for the first time. Existing research has predominantly focused on cognitive performance, biomarkers, or clinical trajectories, or has been limited to documenting the cognitive complaints [36,37,38]. As a result, the broader subjective landscape in which older adults interpret their symptoms at the moment of the first neuropsychological evaluation remains largely underexplored, not only in terms of the level of these psychosocial factors, but also in terms of how they are jointly organized. Importantly, psychosocial dimensions such as perceived memory complaints, attitudes toward aging, aging stereotypes, and subjective age are unlikely to operate independently. Rather, prior research suggests that older adults’ interpretations of aging and memory problems rely on interrelated systems of beliefs, emotions, and self-perceptions operating at different levels. These include symptom-related perceptions of memory complaints, broader attitudes toward aging, socially shared stereotypes about aging, and subjective age as an identity-related marker. While conceptually distinct, these four dimensions are likely to interact and jointly shape how individuals make sense of cognitive changes, particularly in contexts of uncertainty, such as a cognitive evaluation. The present study addresses this gap by documenting, for the first time, the organization of these four psychosocial dimensions in older adults referred for a first diagnostic assessment. By focusing on the structure of associations among these factors, rather than on their isolated levels, this approach is aimed at providing unique insight into the subjective mindset with which older adults arrive at the memory clinic, at a moment when worries, expectations, and representations of cognitive aging are actively being shaped. Rather than focusing solely on the content of these perceptions, the central question concerns their organization: how are beliefs, emotions, and self-perceptions interrelated within this specific clinical context, and how does this organization compare with that observed in a non-clinical setting?

Building on this structural perspective, the present study offers three complementary contributions. First, it provides a detailed descriptive analysis of the structural organization of four key psychosocial dimensions: perceptions of memory complaints, attitudes towards aging, stereotypes of older adults, and subjective age. These dimensions are examined within a cohort of patients undergoing their initial diagnostic assessment in a memory clinic. Second, the study proposes a novel comparison between this clinical patient sample and a control group of community-dwelling older adults who present similar self-reported memory complaints but were recruited from a non-clinical research setting. This design allows us to investigate whether the clinical context, characterized by diagnostic uncertainty, heightened concerns and potential stereotype threat, may be associated with a different configuration of psychosocial variables related to cognitive aging differently than a non-clinical context. While acknowledging that these two populations may differ on other aspects, this comparison highlights how the organization of the psychosocial variables may vary according to the setting in which they are addressed. Third, the study adopts a network analytic approach to the complex structure of memory perceptions and other related constructs. Unlike traditional methods that typically analyze variables in isolation or aggregate them into latent constructs, network analysis conceptualizes the psychosocial variables as an interconnected system [39]. This approach enables the identification of the most influential variables within this system for each [40] and provides a description of their internal organization without imposing an a priori structure.

## 2. Materials and Methods

### 2.1. Sample

Two groups of older adults participated in the study: a patient group and a control group. The patient group consisted of older adults referred for a first diagnostic consultation at a hospital memory clinic, who were recruited during their initial short cognitive evaluation by neurologists from six hospitals in four French cities (Caen, Marseille, Poitiers, and Rouen) via the AGING consortium. The control group consisted of older adults recruited in Marseille and Poitiers through public advertisement to participate in a laboratory-based memory study using the same protocol as in the clinical setting, with inclusion procedures conducted by a neuropsychologist specifically recruited and trained for the study.

Inclusion criteria were identical for both groups. Eligibility was determined based on a standardized clinical interview and participants’ performance on a short battery of cognitive tests and questionnaires: the Mini Mental State Examination (MMSE) [41], the Questionnaire of Cognitive Complaints (QCC) [42], the Geriatric Depression Scale (GDS) [43], the 5-word test [44] and the Instrumental Activities of Daily Living (IADL) [45]. To be included, participants were required to be over 50 years old, report cognitive complaints and meet the following criteria: QCC score ≥ 3, GDS score ≤ 9, MMSE score ≥ 22, 5-word test ≥ 5 and IADL = 0. Participants were excluded if they showed any signs of Alzheimer’s disease according to the NINCDS-ADRDA criteria (National Institute of Neurological and Communicative Diseases and Stroke/Alzheimer’s Disease and Related Disorders Association), had a history or current traumatic brain injury, neurological or cardiovascular disorders, psychiatric disorders (schizophrenia, bipolar disorder or major depression), used psychotropic medication, or reported alcohol abuse. These variables were used as an inclusion criterion and were not used in further analysis.

A total of 130 patients (44 men and 86 women; mean age: 70.99 ± 8.74; age range: 50–87; mean education level: 12.27 ± 3.38 years), and 84 controls (26 men and 58 women; mean age: 66.44 ± 8.09; age range: 50–89; mean education level of 14.44 ± 2.86 years) were included. Descriptive statistics are presented in Table 1. Patients were significantly older, with a mean age difference of five years, and had significantly lower education levels, by around two years, compared to the controls. They also reported more perceived memory difficulties (according to QCC), with a large effect size [46], and they showed lower global cognitive performance (MMSE), with a small-to-medium effect.

### 2.2. Procedure

The present study uses data from a larger project, the longitudinal AGING study [47]. The data analyzed here were collected at the beginning of the protocol, immediately after the inclusion tests (see Table 1 for descriptive statistics) and prior to the next visit during which the first neuropsychological battery—identical for all participants—was administered (results are not presented here). Between these two stages, participants were asked to complete at home a psychosocial questionnaire assessing four domains related to aging and memory: self-perceptions of memory deficits, attitudes toward aging, attitudes toward aging stereotypes, and subjective age. They were instructed to return the completed questionnaire to the hospital or laboratory at the time of their neuropsychological assessment. The present article focuses exclusively on analyses conducted on this psychosocial questionnaire, without taking into account data from subsequent stages of the AGING study.

### 2.3. Measures

Eighteen subscales from different validated instruments were used to operationalize the four psychosocial domains assessed in the questionnaire—self-perceptions of memory deficits, attitudes toward aging, aging stereotypes, and subjective age—while avoiding redundancy across measures (see Table 2 for an overview). As the same questionnaire was administered to both groups, establishing the comparability of responses between patients and controls was essential. Therefore, multi-group confirmatory factor analyses (MG-CFA) were conducted to test measurement invariance. Details of these analyses are provided in the Online Supplementary Material. The results supported the three levels of measurement invariance between the two groups (configural, scalar, and metric invariances), indicating equivalent factorial structures. Consequently, mean scores were computed for each subscale and for each group and used for subsequent descriptive analyses, and internal consistency was estimated using Cronbach’s alpha coefficients (Table 3).

#### 2.3.1. Self-Perceptions of Memory Deficits

Perceptions of memory difficulties were assessed using the French short version of the Illness Perception Questionnaire–Memory (IPQ-M), developed and validated by Besozzi et al. [48] from the original English version by Hurt et al. [5]. This instrument is designed to assess how older adults perceive and emotionally experience their own memory problems. The French short version includes 10 subscales. The first one, *Identity*, comprised 19 items referring to common memory symptoms. For each of them, participants were asked two questions: (1) whether they had ever experienced the symptoms, and (2) whether they believed it was related to their memory problems. The score for this dimension corresponded to the total number of symptoms endorsed as being associated with memory problems.

**Table 2:** Overview of the questionnaire’s variables.

Source	Variable Name	Description	Interpretation of Highest Score
***Perceptions of memory complaints***			
*Illness Perception Questionnaire-Memory (IPQ-M)* [5]	Identity	Number of physical symptoms related to memory problems	More symptoms related to memory problems
	Timeline acute/chronic	Expected duration of their memory problems	Memory problems will persist for a long time
	Timeline stability/decline	Perceived progression of memory	Memory problems will worsen over time
	Personal control (Helplessness)	Perceived ability to prevent/improve memory problems	Stronger perceived control over memory problems.
	Personal control (Blame)	Guilt about not doing enough to prevent memory problems	Stronger feeling of guilt
	Consequences	Negative impact of memory problems on one’s life	Memory problems have a negative impact on life.
	Treatment control	Memory problems can be managed with medical care	Medical treatment can help manage memory
	Emotional representation	Negative emotional response to memory problems	Stronger negative emotional reactions to memory problems.
	Illness coherence	Perceived understanding of one’s memory problems	Greater insight regarding memory problems
	Social comparison	Comparison of one’s memory with that of same-aged peers	Stronger perceptions of having a worse memory than same-aged peers
***Attitudes towards aging***			
*Aging opinion survey* [49]	Personal anxiety	Anxiety and negative emotions about one’s own aging	Greater anxiety toward aging
*Aging Perceptions Questionnaire* [29]	Consequences positives	Beneficial aspect of aging	More positive attitudes toward aging
	Control positive	Positive aspects depend on their own actions	More positive attitudes toward aging
*Fear of Alzheimer’s Disease Scale* [32]	Fear of developing Alzheimer’s disease (AD)	Concerns related to the possibility of developing Alzheimer’s disease	Stronger general fear of developing the disease
***Stereotypes of older adults***			
*Attitudes towards older people* [50]	Mental deterioration	Negative stereotypical beliefs about age-related cognitive decline	Stronger endorsement of negative stereotypes about cognitive aging
***Subjective age***			
*Self-perceptions of aging* [12]	Felt age	Age the person feels	A negative score indicates feeling younger than their chronological age, a positive score indicates feeling older
	Desired age	Age the person wishes to have	
	Apparent age	Age the person believes others attribute to them	

Note. Bold italic text indicates each psychosocial domain. Italic text indicates the names of the instruments used for each variable.

Each of the nine remaining subscales consisted of three items rated on a 5-point Likert scale ranging from 1 (strongly disagree) to 5 (strongly agree). Subscale scores were computed by averaging the three item ratings, after reverse scoring when necessary. The *Timeline acute/chronic* subscale assessed beliefs about the expected duration of their memory problems (e.g., “My memory problems will last for a long time”). Higher scores indicate stronger beliefs that memory problems will persist for a long time. The *Timeline stability/decline* subscale assessed beliefs about the perceived progression of memory (e.g., “My memory problems are likely to become worse with time”), with one item reverse-scored. Higher scores indicate stronger beliefs that memory problems will worsen over time. The *Personal control (helplessness)* subscale assessed beliefs about one’s perceived ability to prevent or improve memory problems (e.g., “What I can do determines whether my memory problems become better or worse”). Higher scores reflect stronger perceived control over memory problems. The *Personal control (blame)* subscale assessed feelings of guilt about not doing enough to prevent memory problems (e.g., “If I made more effort my memory problems would get better”). Higher scores indicate stronger guilt. The *Consequences* subscale assessed beliefs about the negative impact of memory problems on one’s life (e.g., “My memory problems have affected my social life”). Higher scores indicate stronger beliefs that memory problems have a negative impact on life. The *Treatment control* subscale assessed beliefs about whether memory problems can be managed, with two items reverse-scored (e.g., “Doctors can’t do anything to help my memory problems”). Higher scores indicate stronger beliefs that medical treatment can help manage memory problems. The *Emotional representation* subscale assessed the negative emotional response to memory problems (e.g., “My memory problems make me feel anxious”). Higher scores indicate stronger negative emotional reactions to memory problems. The *Illness coherence* subscale assessed perceived understanding of one’s memory problems, with all items reverse-scored (e.g., “I don’t understand why I have memory problems”). Higher scores indicate greater insight regarding memory problems. The *Social comparison* subscale assessed comparison of one’s memory with that of same-aged peers, with one item reverse-scored (e.g., “My memory is worse than other people my age”). Higher scores indicate stronger perceptions of having a worse memory than same-aged peers.

#### 2.3.2. Attitudes Towards Aging

Attitudes toward aging were assessed using four selected subscales from three validated instruments, all rated on a 5-point Likert scale ranging from 1 (strongly disagree) to 5 (strongly agree).

The *Personal anxiety towards aging* subscale of the Aging Opinion Survey (AOS) [49] was used to assess anxiety and negative emotions about one’s own aging. Eleven of the original 15 items were retained (e.g., “The older I become the more I worry about my health”), as four were considered irrelevant to the study’s goal (e.g., “Most older people seem to need a lot of extra sleep to have enough energy for everyday chores”). The subscale score was computed as the mean of the 11 item ratings (after reverse-coding three items), with higher scores indicating greater anxiety toward aging.

The *Consequences positive* and *Control positive* subscales of the Aging Perception Questionnaire (APQ) [29] were used to assess older adults’ positive beliefs about aging. The *Consequences positive* subscale (4 items) measures beliefs about the beneficial aspect of aging (e.g., “As I get older, I get wiser”), while the *Control positive* subscale (5 items) measures beliefs that these positive aspects depend on their own actions (e.g., “Whether I continue living life to the full depends on me”). For each subscale, the score was computed as the mean of the item ratings, with higher scores indicating more positive attitudes toward aging.

*The Fear of Alzheimer’s Disease Scale* (FADS) [32] was used to assess concerns specifically related to the possibility of developing Alzheimer’s disease, a facet not captured by the previous instruments. To address this complementary aspect without placing disproportionate emphasis on this disease, three items were selected from the General Fear subscale of the original questionnaire. This reduced subscale measures general fears of developing Alzheimer’s disease independently of current memory difficulties (e.g., “The older I get, the more fearful I become that I may develop Alzheimer’s disease”). The subscale score was computed as the mean of the three item ratings, with higher scores indicating stronger general fear of developing Alzheimer’s disease.

#### 2.3.3. Aging Stereotypes

Stereotypes about cognitive aging were assessed using the Mental Deterioration subscale of the *Attitudes toward old people scale* [50]. This subscale comprises 14 items assessing negative stereotypical beliefs about age-related cognitive decline (e.g., “They are absent minded”), rated on a 5-point Likert scale ranging from 1 (strongly disagree) to 5 (strongly agree). One additional item, developed specifically for the AGING study by the team, was added (“Elderly people inevitably develop conditions such as Alzheimer’s disease”) to capture Alzheimer’s-related stereotypical beliefs relevant to the study. Subscale scores were computed as the mean of the item ratings, with a higher score indicating stronger endorsement of negative stereotypes about cognitive aging.

#### 2.3.4. Subjective Age

Subjective age was assessed using three questions adapted from [12], capturing three facets of subjective age: *Felt age* (“How old do you feel?”), *Desired age* (“If you could choose your age, how old would you want to be?”) and *Apparent age* (“According to you, how old do people generally think you are?”). Responses were provided in written form. For each subjective variable, a chronological age was accounted for by computing a proportional discrepancy score using the following formula [13]: “(100 × [x − chronological age]/chronological age)”, where x corresponds to each subjective age measure. A negative score indicates that the participant’s subjective age is lower than their actual chronological age (i.e., perceiving themselves as being younger), whereas a positive score indicates the participant feels older. Those discrepancy scores were used as our subjective age variables for subsequent analyses.

### 2.4. Statistical Analysis

All statistical analyses were performed using R studio (version 2024.09.0.375). Since one participant from the patient group failed to submit the questionnaire and was removed from the following analyses, the final sample consisted of 213 participants: 129 in the patient sample and 84 in the control sample. Statistical analyses were divided into four main steps: (1) ANCOVAs, (2) Network analysis, (3) estimation of centrality indices and (4) bootstrap procedures.

Analyses of covariance (ANCOVA) were first performed to compare adjusted means of each subscale between samples, using age as a covariate since it differed significantly between our two samples. Other variables that showed significant differences were not included as covariates. Differences in QCC and MMSE scores between the two samples likely reflect inherent clinical characteristics, as the clinical sample is expected to report greater cognitive complaints and show lower cognitive performance than the non-clinical sample. Therefore, MMSE and QCC were not included as covariates. Education level was not included as a primary covariate in the main analyses, as the central objective of the study was to examine aging-related psychosocial representations rather than to isolate the specific contribution of educational attainment. The potential influence of education was instead examined in supplementary analyses. Spearman’s bivariate correlations were computed to examine the associations between the subscales with statistical significance set at *p* < 0.05. Cronbach’s α was calculated only for subscales measured with 5-point Likert scales. Therefore, it was not computed for the Identity subscale, which consists of a list of symptoms endorsed as being associated with memory problems, nor for subjective age variables (Felt age, Desired age, and Apparent age) given that each captures a distinct idea. Therefore, each of these variables was assessed as a single item and analyzed separately.

In addition to ANCOVAs comparing group differences in mean levels of psychosocial variables, network analyses were conducted to explore the structural organization of four psychosocial dimensions related to aging and memory within each group (i.e., perceptions of memory complaints, attitudes toward aging, stereotypes of older adults, and subjective age). Separate networks were computed for the patient and control groups. These analyses were based on the full set of subscales operationalizing these domains (i.e., all psychosocial variables included in the questionnaire). This approach allowed us to visualize and describe potential differences in the overall structure and organization of psychosocial perceptions between the two samples. Each network was estimated using partial correlations [51]. Additionally, EBIC-lasso networks were estimated. However, due to limited sample sizes in both patient and control groups, the resulting networks were highly sparse, as EBIC-lasso is a strict model and suppressed weaker edges [51], therefore leading to most nodes being isolated from each other. These preliminary results are available from the authors upon request. Therefore, given the small sample sizes and the exploratory descriptive aim of the present study, we decided to opt for partial correlation networks rather than regularized EBIC-lasso networks (which typically requires N > 100 [51,52,53]). Partial correlation networks estimate associations between two variables while controlling for all others, based on the inverse of the correlation matrix, and were used here to characterize the overall organization of psychosocial variables [54]. In the resulting network, each variable (i.e., each subscale of the questionnaire) is represented by a node, and the statistical associations between variables are represented by edges connecting these nodes. The edges are undirected, indicating the presence of an association between two variables without implying any causal direction. Nodes are placed in the network based on the spring layout [54], which puts each node according to the strength and number of connections: variables that are more strongly or more frequently connected appear closer together. Moreover, communities in the networks have been estimated based on random walks using the Walktrap method [55].

Once the networks were estimated, we computed two centrality indices to identify which variables were of importance within each structure. First, *Strength* centrality reflects how strongly a node is connected to others by computing the sum of absolute edge weights from each node. *Expected influence* complements *Strength* centrality by accounting for both positive and negative connections (edges), reflecting whether changes in a node might enhance or inhibit effects within the network [40,51]. Both indices were standardized as z-scores with mean = 0 and SD = 1. Nodes were then interpreted as follows: for both Strength and Expected Influence, nodes with a z-score around 0 were considered as very low strength/influence in the network, nodes with z between 0 and +/−1 as moderately above average, and nodes with z ≥ |1| (i.e., ≥1 SD above the mean) as highly strong/influential. While these thresholds are not standard in the psych-network literature, they align with conventional interpretations of z-scores and facilitate interpretability.

Finally, a bootstrap procedure was conducted to assess the stability of the centrality indices using correlation stability coefficients (CS-coefficients) [51]. Both indices showed good robustness in both samples exceeding the recommended threshold of 0.25. Specifically, a value of 0.682 was obtained for Strength and 0.767 for Expected Influence in the patient sample, and a value of 0.393 for Strength and of 0.631 for Expected Influence in the control sample. Therefore, both networks can be considered stable.

### 2.5. Data Preparation

All data preparation procedures were guided by psychometric criteria and applied consistently across groups to ensure the reliability and interpretability of subsequent analyses.

Among the study scales, most showed good internal reliability with Cronbach’s α. Two exceptions were *Personal control (Helplessness)* (0.55 for patients and 0.57 for controls) and *Treatment control* (0.56 for patients and 0.58 for controls). Because these values fall below acceptable reliability thresholds [56], both subscales were removed for further analysis, as such low reliability would introduce substantial measurement error into network estimation [57]. By contrast, *Consequences* and *Illness coherence* showed low internal consistency in the patient sample (α = 0.58 and 0.59, respectively), but good reliability in the control sample. Although reliability was lower in patients, these dimensions are theoretically central to the study of illness and aging representations and excluding them would have resulted in a substantial loss of construct coverage. Their inclusion was therefore considered acceptable within an exploratory network framework, while acknowledging the potential impact of measurement noise. Finally, both *Timeline stability/decline* and *Illness coherence* initially showed low α values as one reverse-coded item was reported to be misunderstood by participants in both samples (based on standardized feedback collected during data acquisition). Removing these items improved reliability, and the items were excluded from subsequent analyses.

Overall, the proportion of missing data was low (1.77%). The randomness of missing values was assessed using the generalized Little’s Missing Completely at Random (MCAR) test [58], which indicated that missing data were randomly distributed across the dataset. For ANCOVA analyses, outliers in the subjective age variables (*Felt age, Desired age* and *Apparent age*) were identified using Tukey’s rule, and values exceeding the lower and upper bounds were removed (<4% of the sample) as extreme values can disproportionately influence mean-level comparisons. Given the MCAR pattern of missingness, the low proportion of missing data, and the focus of ANCOVA on mean-level comparisons, cases with missing values were removed using listwise deletion. In contrast, for the network analysis, which rely on correlation matrices and are more sensitive to missing data and sample size fluctuations, missing values were imputed using Multiple Imputation by Chained Equations (MICE) on each group separately. Consequently, correlation and network analyses were conducted using the imputed datasets, ensuring stable estimation of associations among variables.

## 3. Results

### 3.1. Descriptive Statistics

Means, standard deviations, Cronbach’s alpha coefficients, and *p*-values derived from one-way ANCOVAs controlling for age for both samples are presented in Table 3. Significant mean differences were observed. Compared with controls, patients endorsed a greater number of symptoms associated with memory problems, perceived these problems as having greater consequences on their lives, and reported stronger emotional reactions to their memory difficulties. Patients also perceived their memory to be worse than that of their same-aged peers and expressed greater fear of developing Alzheimer’s disease compared to controls. The largest effects were observed for *Consequences* and *Emotional representation*.

**Table 3:** Descriptive statistics for all variables of the questionnaire for patients and control samples.

	Patient Group (Hospital) *n* = 129		Control Group (Laboratory) *n* = 84		*p*-Value	η^2^ *p*
	Mean (±SD)	Cronbach’s α	Mean (±SD)	Cronbach’s α		
**Self-perceptions of memory deficits**						
Identity	8.04 (±4.37)	-	6.79 (±4.54)	-	**0.048**	0.02
Timeline acute/chronic (Time_a/c)	3.55 (±0.93)	0.78	3.70 (±0.94)	0.71	0.250	0.01
Timeline stability/decline (Time_s/d)	3.79 (±0.93)	0.88	3.79 (±0.94)	0.84	0.990	0.00
Personal control (Blame)	3.29 (±0.91)	0.78	3.66 (±0.94)	0.73	**0.005**	0.04
Consequences (Conseq)	2.85 (±0.89)	0.58	2.08 (±0.90)	0.72	**<0.001**	0.15
Emotional representation (Emo_Rep)	3.31 (±1.15)	0.86	2.45 (±1.15)	0.83	**<0.001**	0.12
Illness coherence (Ill_Coh)	2.68 (±1.03)	0.59	3.51 (±1.04)	0.72	**<0.001**	0.13
Social comparison (Soc_Comp)	2.99 (±0.92)	0.76	2.42 (±0.93)	0.77	**<0.001**	0.08
**Attitudes toward aging**						
Personal anxiety towards aging (Anxiety)	2.89 (±0.75)	0.73	2.71 (±0.75)	0.57	0.091	0.01
Consequences positive (Conseq_pos)	3.63 (±0.85)	0.68	3.57 (±0.86)	0.76	0.641	0.00
Control positive (Ctrl_pos)	4.33 (±0.66)	0.78	4.41 (±0.66)	0.66	0.406	0.00
General fear (Gen_Fear)	2.86 (±0.97)	0.65	2.48 (±0.99)	0.66	**0.008**	0.04
**Stereotypes of older adults**						
Mental deterioration (Men_Det)	2.44 (±0.58)	0.84	2.44 (±0.59)	0.84	0.994	0.00
**Subjective age**						
Felt age	63.10 (±8.23)	-	61.20 (±8.44)	-	0.116	0.01
Discrepancy score for Felt age (Felt_a)	−8.98 (±11.81)	-	−11.68 (±12.10)	-	0.114	0.01
Desired age	47.60 (±14.54)	-	45.40 (±14.39)	-	0.288	0.00
Discrepancy score for Desired age (Des_a)	−30.80 (±20.67)	-	−34.00 (±20.35)	-	0.277	0.00
Apparent age	62.00 (±4.99)	-	62.10 (±4.87)	-	0.846	0.00
Discrepancy score for Apparent age (App_a)	−10.06 (±7.16)	-	−9.76 (±6.97)	-	0.766	0.00

Note. Bold text indicates each psychosocial domain. *p*-values for means comparison were obtained with one-way ANCOVAs, controlled for age. SD = Standard Deviation.

In contrast, the control group reported a greater feeling that they did not do enough to prevent memory problems compared to patients. They also reported better insight into their memory problems. The largest effect was shown for *Illness coherence*.

Regarding subjective age variables, no significant differences were observed between groups. In fact, both patients and controls reported feeling younger that their chronological age, believed that others perceive them as younger and expressed a desire to be younger.

### 3.2. Correlation Matrix

**Figure 1:**
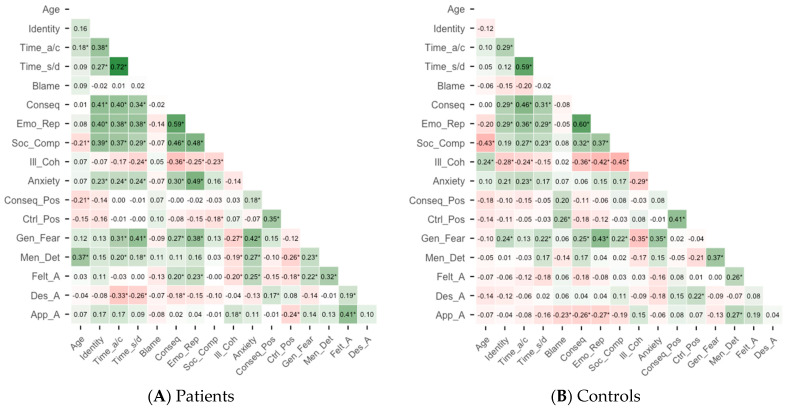
Correlations for all psychosocial variables for patients (Panel (**A**)) and controls (Panel (**B**)). Note. Correlation coefficients range from −1 (in red) to 1 (in green). * *p* < 0.05. Age: Chronological age; Identity: Identity; Time_a/c: Timeline acute/chronic; Time_s/d: Timeline stability/decline; Blame: Personal control (Blame); Conseq: Consequences; Emo_Rep: Emotional representation; Ill_Coh: Illness coherence; Soc_Comp: Social comparison; Anxiety: Personal anxiety towards aging; Conseq_Pos: Consequences positive; Ctrl_Pos: Control positive; Gen_Fear: General fear; Men_Det: Mental deterioration; Felt_A: Felt age; Des_A: Desired age; App_A: Apparent age.

The heatmap displaying the correlation matrices of the questionnaire variables is presented in Figure 1. A greater number of positive correlations (green) are observed in the patient sample, particularly between variables reflecting negative emotions: e.g., self-perceptions of memory deficits (i.e., correlations with *Emotional Representation*), the aging process (i.e., correlations with *Mental Deterioration*), or with variables related to subjective age (i.e., correlations with *Apparent Age*). In contrast, the control sample shows a greater number of negative correlations (red) among those variables, suggesting more differentiated or protective attitudinal patterns. The chronological age variable does not correlate with the three subjective age measures probably because the calculation of the discrepancy scores already accounted for, and thus removed, the variance associated with chronological age differences within the samples. Despite these overall differences in correlation patterns, some associations are shared across groups. In both samples, illness coherence, for instance, shows moderate-to-strong negative correlations with negative emotional variables (i.e., *Consequences, Emotional Representation* and *Social Comparison*).

### 3.3. Network Analysis

**Figure 2:**
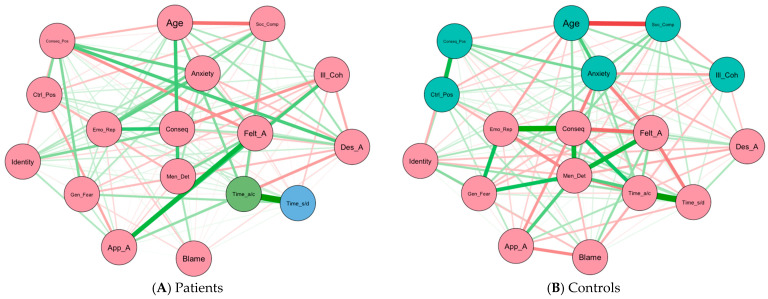
Network of all psychosocial variables of the AGING questionnaire in (Panel (**A**)) patients and (Panel (**B**)) controls. Note. Green lines indicate positive connections; red lines indicate negative connections; thickness of the edge represents its strength. Node colors represent the different clusters identified through community detection analyses. Age: Chronological age; Identity: Identity; Time_a/c: Timeline acute/chronic; Time_s/d: Timeline stability/decline; Blame: Personal control (Blame); Conseq: Consequences; Emo_Rep: Emotional representation; Soc_Comp: Social comparison; Ill_Coh: Illness coherence; Anxiety: Personal anxiety towards aging; Conseq_Pos: Consequences positive; Ctrl_Pos: Control positive; Gen_Fear: General fear; Men_Det: Mental deterioration; Felt_A: Felt age; Des_A: Desired age; App_A: Apparent age.

Figure 2A,B display the network models estimated for the patient and control samples, respectively. From a visual inspection, both networks share some similarities in patterns of the associations. For example, both networks show negative correlations between *Age* and *Social Comparison*, and positive ones between *Timeline acute/chronic* and *Timeline stability/decline*. Despite these shared patterns, the organization of the two networks appears to differ.

First, although the variables in both networks displayed similar patterns of links, the direction of these associations (whether the connection was positive or negative) and their magnitude differed between groups. In the patient sample, partial correlations are predominantly positive, whereas in the control sample they tend to be negative. For example, subjective age variables (*Felt*, *Desired*, and *Apparent age*) are linked to similar self-perceptions of memory deficits variables in both networks, but with opposite signs. *Felt age* and *Personal anxiety towards aging* are linked positively in the patient sample, whereas this association is negative in the control sample. Similarly, *Apparent age* shows a positive association with *Timeline stability/decline* in patients, but a negative one in controls. These patterns suggest that, although the same variables are involved, their interrelations are structured differently across the two groups.

Second, beyond differences in directionality, both networks show strong partial correlations among variables related to attitudes toward aging, subjective age, and self-perceptions of memory deficits, but relying on different variables within each domain to structure these associations. In the patient sample, stronger partial correlations are observed between *Felt age* and *Illness coherence*, or between *Personal anxiety towards aging* and *Emotion representation*, compared to these links in the control sample. In contrast, the control sample exhibits stronger associations among a different set of variables, such as correlations between *Felt age* and *Consequences* or *Personal anxiety towards aging* and *Apparent age*, compared to patients. The control sample also shows stronger correlations between stereotypes of older adults (*Mental deterioration*) and other variables (i.e., *General fear, Timeline acute/chronic, Personal anxiety towards aging*) compared to the patient sample. These connections indicate that although self-perceptions of aging are associated with negative emotional components and threat-related beliefs in both groups, the configuration of these links differs. At the same time, in the control sample, these threat and emotion-related variables are more negatively linked to variables with a more control-related function such as *Personal control (Blame)*, *Desired age*, and *Illness coherence*. These patterns suggest that, despite their higher presence in the control group, other variables may dampen their impact.

**Figure 3:**
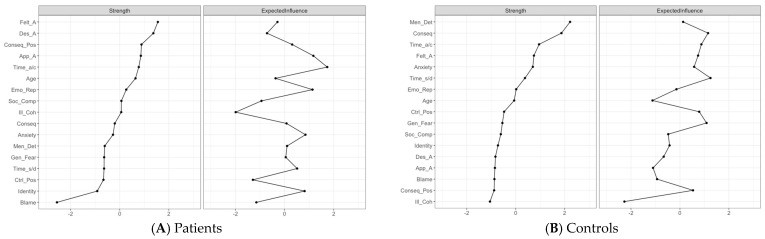
Centrality indices of all psychosocial variables of the AGING questionnaire in patients (Panel (**A**)) and controls (Panel (**B**)). Strength reflects the overall degree of connectivity to each node, identifying the structural hubs of the network. Expected Influence (EI) complements this by including the sign of each edge. A positive EI indicates that a variable tends to reinforce the activity of connected nodes whereas a negative EI indicates a buffering effect on connected nodes. Note. Age: Chronological age; Identity: Identity; Time_a/c: Timeline acute/chronic; Time_s/d: Timeline stability/decline; Blame: Personal control (Blame); Conseq: Consequences; Emo_Rep: Emotional representation; Ill_Coh: Illness coherence; Soc_Comp: Social comparison; Anxiety: Personal anxiety towards aging; Conseq_Pos: Consequences positive; Ctrl_Pos: Control positive; Gen_Fear: General fear; Men_Det: Mental deterioration; Felt_A: Felt age; Des_A: Desired age; App_A: Apparent age.

In addition, these organisational differences are also reflected in the relative importance of nodes within each network (i.e., centrality indices). Figure 3A,B display the centrality indices—Strength and Expected Influence—for each node in the patient and control samples, respectively. These indices reveal differences in the nodes that play a more central role in each network.

In the patient network, *Felt age* shows the highest Strength value, followed by *Desired age*. As visually represented in Figure 2A, *Felt age* is strongly linked to *Apparent age, Illness coherence, Consequences positive* and *Social comparison*, indicating that subjective age variables occupy a central position within patients’ perceptions of memory decline and aging. The least central nodes were *Personal control (Blame)*, followed by *Identity*, which were on the periphery of the network. Expected Influence (EI) provides a complementary functional perspective by showing how these central nodes relate to the rest of the network through positively or negatively valenced connections. The most influential node according to EI (i.e., having the largest number of positive connections) is *Timeline acute/chronic,* followed by *Apparent age* and *Emotional representation*. These variables are associated with self-perceptions of memory deficits, stereotypes of older adults, and subjective age. Conversely, the node with the most negative EI value is *Illness coherence*. This node represents insight into one’s memory problems; which reflects greater perceived understanding of one’s memory problems.

A different pattern emerges in the control network. The nodes with the highest Strength are *Mental Deterioration* and *Consequences*. As visually represented in Figure 2B, both variables are strongly linked with negative emotional components and threat-related beliefs, indicating that endorsement of negative stereotypes about cognitive aging and negative emotional reactions to memory problems constitute central components of controls’ self-perceptions of aging. Conversely, *Illness Coherence* shows the lowest Strength value, suggesting that insight regarding memory deficits is less integrated within controls’ representational system. Regarding EI, the most influential nodes in the control network are *Timeline stability/decline*, *Consequences*, and *General Fear*. These variables are closely associated with negative emotional and threat-related variables. However, as in the patient sample, the most negatively influential node is again *Illness coherence*.

Finally, community detection analyses further highlight structural differences between the two samples by identifying different clusters (i.e., communities of closely related nodes) in each group. In the patient network, no clear, well-separated clusters are detected. A dominant cluster encompasses nearly all variables, with the exception of *Timeline acute/chronic* and *Timeline stability/decline*, which are identified as isolated communities. In contrast, the control network partitions into two distinct clusters. The larger cluster (11 nodes) encompasses variables centered around *Mental deterioration*, including all subjective age variables and most negative emotional components and threat-related beliefs. Meanwhile, the smaller cluster (6 nodes) groups the remaining variables, primarily composed of positive views of aging (*Consequences positive*) and control-related variables (*Illness coherence* and *Control positive*), suggesting a more segmented organization of aging-related perceptions in the control network compared to the patient network.

In addition, because education attainment is known to influence both perceived vulnerability and cognitive performance [59,60], we conducted supplementary ANCOVA and network analyses including years of education as an additional covariate. These analyses yielded results that were qualitatively identical to those obtained when controlling for age only. Given our focus on aging-related processes, we therefore retained the original model including age as the sole covariate. Detailed results from the education-adjusted analyses are reported in the Supplementary Materials (Table S4 and Figures S1–S3).

## 4. Discussion

The present study aimed to explore the structural organization of psychosocial self-representations related to aging and memory in older adults undergoing a first diagnostic evaluation in a memory clinic, compared with community-dwelling older adults assessed in a non-clinical setting. This comparative approach was designed to capture how distinct assessments contexts shape the organisation of these self-representations. Rather than determining which group performs better or worse, our goal was to examine how psychosocial representations are structured differently across meaningful clinical and non-clinical settings. Using a network approach, we investigated how psychosocial dimensions including perceptions of memory complaints, attitudes toward aging, aging stereotypes, and subjective age are interconnected, how they are organized into broader structures, and which variables occupy more central positions within these networks. By comparing network configurations across clinical and non-clinical contexts, the study characterized how the organization of psychosocial representations differs depending on whether memory complaints are experienced within a diagnostic setting.

Overall, our findings revealed that although both groups share partially similar patterns of associations, their representational systems are organized around distinct central variables. This suggests different ways of interpreting cognitive difficulties in the two groups which may be attributed to whether individuals are confronted or not to a clinical evaluation. Specifically, the patient network is primarily organized around indicators of subjective age, whereas the control network is centered around the internalization of aging stereotypes. This divergence highlights the importance of contextual factors, particularly the initial diagnostic evaluation, in shaping the organization of psychosocial representations of aging and memory.

### 4.1. Chronicity Beliefs and Subjective Age as Influencers of Emotional Distress in Patients

Overall, the patient network shows a configuration consistent with a clinical context marked with uncertainty and perceived threat. Attending a memory clinic for a first diagnostic evaluation confronts older adults with the possibility of receiving a diagnosis and raises concerns about future cognitive decline, a situation likely to heighten vulnerability and emotional distress. Within this network, *Timeline acute/chronic* emerges as the node with the highest expected influence. This dimension captures beliefs about whether memory difficulties are perceived as temporary or long-lasting, and by extension, reflects their perceived severity. In the context of a first diagnostic evaluation, such beliefs are particularly salient, as patients face uncertainty regarding whether their symptoms reflect transient difficulties or a progressive condition. Concerns about chronicity may therefore reflect fears related to disease progression and anticipated life changes associated with a potential diagnosis [15]. Consistent with this, *Timeline acute/chronic* is positively linked to several negative emotional components, indicating that perceiving memory complaints as chronic is embedded within a broader pattern of emotional distress and negative self-perceptions of aging.

The second most influential node in the patient network is *Apparent age*, reflecting how individuals believe they are perceived by others (e.g., family, friends, healthcare professionals). This variable may be particularly relevant in a clinical context, as help-seeking is often initiated by the social environment rather than the patients themselves [18,19,61]. The negative correlation between *Apparent age* and *Control positive* suggests that being perceived as younger by others is linked to a stronger sense of control over memory decline. This may imply that patients rely on external feedback to evaluate their own aging and cognitive difficulties. Importantly, *Apparent age* is strongly and positively correlated with *Felt age*, indicating that internal experiences of aging are closely related to perceived external judgements. While *Apparent age* reflects perceived social judgements, *Felt age* integrates these external cues with subjective experiences, which may explain its emergence as the most central node. *Felt age* is positively linked to several negative representations of aging, including *Personal anxiety towards aging, Consequences* or *Mental deterioration*, suggesting that feeling older is closely intertwined with emotional distress and threat-related interpretations of aging and memory decline in the clinical context.

Together, these results highlight that subjective age occupies a central position within patients’ psychosocial representational system, linking emotional reactions, beliefs about aging, and interpretations of memory difficulties within the clinical context. Rather than operating as an isolated perception, subjective age appears embedded in a broader system through which patients make sense of their cognitive difficulties under conditions of diagnostic uncertainty. This interpretation is consistent with previous research identifying subjective age as a key marker of aging quality [34], with younger subjective age associated with better health outcomes and greater engagement in health-promoting behaviors [33]. In line with this literature, the present findings suggest that feeling younger may serve a protective function by attenuating negative emotional representations, whereas feeling older may amplify vulnerability in stressful or threatening contexts [13,62]. Likewise, the negative association between *Felt age* and *Consequences positive* indicates that feeling younger is associated with patients’ beliefs in the possibility of positive outcomes in the aging process. As no significant differences were observed between groups for any subjective age variables, their relevance in the patient group appears to derive from their integration in broader psychosocial representational systems, as revealed by the network analyses.

Taken together, these results suggest that patients mostly rely on subjective age, integrating both internal feelings and perceived external judgements, to interpret their cognitive difficulties and aging process in a clinical context.

In addition, *Emotional representation* emerges as a third influential node, capturing overall negative emotional responses towards memory deficits. This node is positively associated with other negative components (e.g., *Personal anxiety towards aging*, *Consequences*), suggesting that negative affect co-occurs with the other negative components. Patients also reported significantly higher levels of emotional distress compared to controls, reinforcing the idea that *Emotional representation* plays a central role, aligning with the emotional distress and perceived threat that can be present in the clinical context. Overall, the patient network reveals a tightly interconnected configuration in which beliefs about symptom chronicity, subjective age, and emotional responses mutually reinforce one another. This pattern is consistent with a representational system shaped by uncertainty, perceived threat, and anticipation of diagnosis, and highlights how the clinical context may foster a dense and emotionally charged organization of psychosocial representations related to aging and memory.

### 4.2. Internalizing Aging Stereotypes in the Control Sample

In contrast to patients, the control network was primarily organized around *Mental deterioration*, which displays the highest strength value. This indicates that the internalization of negative aging stereotypes occupies a central position in controls’ representations of aging and memory. *Mental deterioration* clustered with several negative emotional components through positive correlations, suggesting that stronger endorsement of aging stereotypes co-occurs with heightened negative emotional responses to memory complaints and aging-related concerns [10].

Notably, in the control network, *Mental deterioration* seems to structure the relationship between subjective age variables, as the association between *Apparent age* and *Felt age* is embedded within a broader configuration centered on aging stereotypes. This contrasts with the patient network, where the association between these two variables is more direct. This pattern suggests that, unlike patients, controls may rely on aging stereotypes as an interpretative framework through which they make sense of how old they look, how old they feel, and how they interpret their memory difficulties.

In line with this interpretation, the positive association between *Mental deterioration* and *Apparent age* suggests that stronger identification with aging stereotypes is linked to being perceived as older by others. In turn, *Apparent age* is negatively associated with *Personal control (Blame)* and *Consequences*, a pattern not observed in the patient network. This configuration suggests that, in controls, appearing older is associated with reduced self-blame and weaker beliefs in severe negative consequences of memory difficulties. Altogether, these results point to controls being more likely to attribute their memory deficits to aging to “normalize” these experiences. In this context, *Felt age* may serve as a protective or normalizing function in controls, allowing them to interpret their memory difficulties within an age-related narrative rather than a personal failure or pathological decline. This interpretation is consistent with the non-clinical setting of the control group, in which cognitive complaints are less severe and not accompanied by an immediate diagnostic threat. In this context, there is little motivation to distance oneself from aging by feeling younger. By contrast, in our patient sample, feeling younger may come as an avoidant coping strategy to distance themselves from the perceived threats associated with aging and potential pathology [63].

Additionally, three other nodes appeared as being important: *Timeline stability/decline*, reflecting beliefs about symptoms progression; *Consequences*, representing beliefs about the negative impact of memory deficits; and *General fear*, referring to fear of developing Alzheimer’s disease. These variables cluster together with other negative emotional components through positive correlations, indicating coherent threat-related beliefs. However, their overall impact appears attenuated compared with patients, as both *Consequences* and *General fear* are reported at significantly lower levels in controls. This pattern is consistent with the less threatening and anxiety-provoking nature of the non-clinical context.

Taken together, these findings suggest that controls rely on aging stereotypes as a central interpretative framework through which memory difficulties are normalized, emotional threat is regulated, and cognitive complaints are integrated into an age-related, rather than pathological narrative.

### 4.3. Illness Coherence as a Regulator of Emotional Distress Across Groups

Across both networks, *Illness Coherence* emerges as the node with the most negative expected influence, suggesting that greater insight into one’s memory difficulties is systematically associated with lower emotional distress. In the control network, *Illness coherence* is the most peripheral node in terms of strength, yet shows predominantly negative correlations, especially with negative emotional components. This pattern suggests that greater insight into one’s memory difficulties is linked to reduced emotional burden. Controls also report significantly higher levels of *Illness coherence* than patients, consistent with previous research indicating that greater insight is associated with lower distress, increased use of coping behaviors, and greater help-seeking [14].

In contrast, within the patient network, *Illness coherence* shows fewer associations with emotional components. This suggests that, in a clinical context marked by uncertainty and perceived threat, insight into memory difficulties may play a less central but still potentially protective role in regulating emotional responses. Importantly, lower levels of *Illness coherence* observed in patients are consistent with the literature, as reduced insight can be considered a feature of cognitive impairment itself, particularly in the context of emerging pathology [18,61]. Taken together, these findings suggest that *Illness coherence* appears to play a similar regulatory role across groups by dampening emotional distress associated with memory complaints. However, this function appears more integrated and effective in non-clinical contexts, whereas in clinical settings, insight may be partially disrupted by diagnostic uncertainty. This pattern supports the idea that insight may operate more strongly at earlier stages of memory deficits, facilitating emotional regulation and adaptive coping behaviors before memory difficulties become salient enough to prompt clinical referral.

### 4.4. Community Structure of Representational Networks

Community detection analyses further highlight structural differences between the two groups. In the control network, two distinct and well-defined clusters emerge. The first one is centered on negative emotional responses and aging stereotypes, while the second cluster encompasses variables related to control and more positive views of aging. This segmented organization suggests a relatively coherent representational system, in which negative and positive dimensions of aging and memory are differentiated and organized into distinct subsystems. Such a structure may partly reflect self-selection processes, as individuals volunteering for research in a non-clinical setting may already be engaged in reflecting on, and attempting to make sense of, their cognitive changes, which also seem to be quite homogeneous.

In contrast, no clear or well-separated clusters emerge in the patient network. Instead, most variables form a single cluster. This lack of clustering may reflect the heterogeneity in patients’ profiles, including variability in severity, etiology, and stage of cognitive decline. It may also indicate that some patients might not have yet developed a coherent or differentiated representation of their memory difficulties and views of aging, particularly when help-seeking is externally initiated. The clinical context itself, characterized by uncertainty, perceived threat, and anticipation of diagnosis [64], may further contribute to a more diffuse and emotionally charged organization of psychosocial representations. Therefore, this may have increased perceived stress, which consequently increased the covariance between variables and artificially inflated the network density. Moreover, this perceived diagnosis threat present in our patients may have acted as an anxious lens through which they responded to the questionnaire. This emotional salience may have played a role in grouping variables together, different from what could be observed outside of the clinical context, in a less stressful environment.

### 4.5. Clinical Impact of Subjective Age and Illness Coherence in Patients

The centrality of subjective age in the clinical group suggests that, apart from being the most important node in the network, it could serve as a valuable target for clinical interventions. Specifically, integrating a conversation between clinicians and patients around subjective age during neuropsychological assessments could allow the clinician to navigate through the emotional distress of the patients and tailor their communication styles to de-emphasize their negative feelings towards aging and memory complaints. By identifying how old a patient feels, the clinician would be able to better predict their reactions to the tests, the diagnosis, the following care, and even improve their overall psychological well-being. In fact, clinical dialogue focused on reducing subjective age could foster a sense of empowerment and help organize more personalized care, as a lower subjective age does not equal denial but helps adopt a more positive view of the aging process and memory deficits, which contribute to their resilience towards the aging symptoms [13,33,62]. Given the interconnected nature of the self-representations of aging, such a shift in subjective age could trigger a cascading effect, resulting in a potential enhancement of their engagement in health-promoting behaviors or their personal disease management. And indeed, some research showed positive effects of a psychological intervention that reframed their subjective age (e.g., by combating aging stereotypes) or reduced fear and negative beliefs about memory decline led to significant decreases in anxiety, self-reported memory failures, depression, and increased social engagement and well-being [65,66,67].

Furthermore, our findings suggest that insight might play a regulatory role in patients. Therefore, they may also benefit from interventions designed to enhance their awareness and knowledge regarding their cognitive complaints. A clearer understanding of their cognitive complaints could lead to a more accepting stance towards their difficulties, deepening the motivation to seek and adhere to appropriate care. Indeed, research shows that increasing literacy about a disease and patient education on the matter improves adherence to treatment, well-being and overall health outcomes [68,69].

### 4.6. Limitations

Several limitations should be acknowledged.

First, the relatively small and unequal sample sizes may have affected the network estimations. Although network analysis can provide meaningful insights even in modest samples, results should be interpreted with caution. Future studies with larger samples would allow for more robust network estimation, including the use of stable EBIC-lasso networks, and allow for formal statistical comparisons between networks (similarly to previous work [70,71]).

Second, the exclusion of control-related constructs, Personal control (Helplessness) and Treatment control, may have influenced the observed network estimations. By removing these two constructs, the relative influence of the remaining variables might have increased, particularly in the patient network, where such variables can play a certain role. Specifically, the reduction in control-related variables in the network may have eliminated links that would otherwise counterbalance self-representations of aging, thereby shifting the network from a coping-oriented structure (e.g., how patients manage memory complaints and aging-related processes) to a more interpretative one (e.g., how patients feel about these experiences). Furthermore, the exclusion of these variables may have prevented other clusters from emerging in the patient network. Likewise, retaining Consequences and Illness Coherence, despite their modest reliability, may have contributed to highlighting clinically meaningful illness-representation nodes, although their measurement noise could also attenuate their connectivity and centrality.

Third, the cross-sectional design of the study captures self-perceptions of aging at a single time point. As a result, the observed networks likely represent transient structural organizations that may evolve over time as cognitive concerns progress or as individuals move through different stages of the diagnostic process. Prior research has shown that constructs such as subjective age are dynamic and can fluctuate daily depending on contextual and emotional factors [34]. Longitudinal studies are therefore needed to examine whether and how these organizations rearrange over time, as cognitive decline progresses. Furthermore, due to the correlational nature of the network analysis, results presented here, particularly centrality indices, should not be interpreted as indicators of causality. Instead, the observed structures provide insight into the representational organization of both patients and controls at a specific point in time and within a particular context (clinical context, while awaiting a potentially life-altering diagnosis vs. non-clinical context, with no subsequent diagnosis). While these networks do not explain the underlying functional mechanisms of the representational system, they do provide valuable information on how variables are interrelated within our samples and lay the ground for further translational research (e.g., experimental studies of interventions that target central nodes identified.

Fourth, the patient group is likely heterogeneous with respect to the nature, severity, and etiology of memory complaints, as well as eventual diagnoses. This heterogeneity may have contributed to the absence of defined clusters in the patient network. Future research could address this limitation by focusing on more homogenous clinical subgroups, for instance based on clinical diagnoses or similar memory complaints, to eventually capture the differences in structural organization.

Fifth, the two groups significantly differed not only in clinical context but also in age, years of education, cognitive performance (MMSE), and severity of subjective cognitive complaints (QCC). Specifically, the clinical context may have induced higher stress due to the hospital setting, or the “diagnostic threat” inherent to the clinical evaluation process [64]. This situational anxiety may have altered the perceived vulnerability in patients, compared to the control group, who were examined in a more neutral setting. MMSE and QCC were not included as covariates because they reflect core clinical characteristics that distinguish patients referred to a memory clinic from non-clinical participants. Partialing out these variables would have altered the very contrast the study aimed to characterize. Education level was examined separately in supplementary network analyses. Importantly, controlling for education did not meaningfully alter the network structure, centrality patterns, or community organization, supporting the robustness of the main findings with respect to educational differences between groups. Nevertheless, as the present analyses are cross-sectional and context-dependent, the observed network structures should be interpreted as reflecting psychosocial configurations specific to the clinical and non-clinical assessment contexts rather than as fixed or causal mechanisms.

Finally, our study was conducted in France; therefore, our entire sample fits within the French culture and healthcare context, which might differ from other countries. For instance, several countries did not have, until recently, a national dementia strategy, unlike France [72]. Additionally, the sample comprises older adults characterized by mild cognitive impairment and intact daily functioning. Therefore, caution is advised when generalizing these findings to other cultural environments or different clinical populations.

Despite these limitations, the present study offers a novel perspective on the interrelations between several psychosocial factors, shaping the subjective experience of aging and memory complaints.

## 5. Conclusions

Taken together, these findings display that older adults rely on different psychosocial variables to make sense of their memory difficulties depending on the context in which these difficulties are experienced. In a clinical context, older adults undergoing a first diagnostic evaluation appear to primarily rely on subjective age, integrating feelings and perceived external judgements, to interpret memory complaints in a context of uncertainty and perceived threat. In contrast, in a non-clinical setting, older adults tend to rely more on aging stereotypes and illness-related beliefs to normalize memory difficulties and regulate emotional responses. By highlighting differences in central variables, network organization, and community structure, this exploratory network analysis underscores the importance of considering psychosocial representations as interconnected systems rather than isolated factors. Such an approach offers valuable insight into the subjective mindset with which individuals approach memory assessment and may help clinicians better understand patients’ expectations, concerns, and vulnerabilities at the critical stage of first diagnostic evaluation.

## Figures and Tables

**Table 1:** Descriptive statistics for inclusion measures in the patient and control groups.

	Patient Group (Hospital) *n* = 130	Control Group (Laboratory) *n* = 84	*p*-Value	Cohen’s *d*
Gender ratio (M/F)	44/86	26/58	0.771 ^1^	--
Age (years) (±SD)	70.96 (±8.74)	66.44 (±8.09)	**<0.001 ^2^**	0.53
[Age range]	[50–87]	[50–89]	-	--
SPC ratio (low/med/high)	44/49/37	21/30/33	0.202 ^1^	--
Education years (±SD)	12.27 (±3.38)	14.44 (±2.86)	**<0.001 ^2^**	0.68
MMSE scores (±SD)	27.45 (±1.74)	28.14 (±1.61)	**0.0042**	0.41
QCC scores (±SD)	5.10 (±1.33)	3.16 (±1.57)	**<0.001 ^2^**	1.36
GDS scores (±SD)	6.19 (±2.71)	5.65 (±3.26)	0.191 ^2^	0.18
5-word test (±SD)	9.95 (±0.26)	10.00 (±0.00)	0.058 ^2^	0.27
IADL scores (±SD)	0.00 (±0.00)	0.00 (±0.00)	-	--

Note. Bold *p*-values are significant with statistical significance set at *p* < 0.05. F = Female, M = Male; SD = Standard Deviation. ^1^ Chi-squared test; ^2^ independent sample *t*-test; SPC—Socio-professional category; MMSE—Mini Mental State Examination; QCC—Questionnaire of Cognitive Complaints; GDS—Geriatric Depression Scale.

## Data Availability

The data presented in this study are available on request from the corresponding author as this study is currently ongoing.

## Supplementary Materials

The following supporting information can be downloaded at: https://www.mdpi.com/article/10.3390/brainsci16020204/s1. (1) Confirmatory factor analysis [5,12,29,32,49,50]; (2) Measurement invariance [73,74,75]; (3) Results [76,77], Table S1: MG_CFA results for Self-perceptions of memory deficits, Table S2: MG-CFA results for Attitudes towards aging, Table S3: MG-CFA results for Aging stereotypes; (4) Recruitment materials; (5) Education-controlled ANCOVA and network analysis, Table S4: Descriptive statistics for all variables of the questionnaire for patients and control samples, Figure S1: Correlations for all psychosocial variables for patients (Panel (A)) and controls (Panel (B)), Figure S2: Network of all psychosocial variables of the AGING questionnaire in (Panel (A)) patients and (Panel (B)) controls, Figure S3: Centrality indices of all psychosocial variables of the AGING questionnaire in patients (Panel (A)) and controls (Panel (B)).

## Abbreviations

The following abbreviations are used in this manuscript:

MMSE	Mini Mental State Examination
QCC	Questionnaire of Cognitive Complaints
GDS	Geriatric Depression Scale
IADL	Instrumental Activities of Daily Living
NINCDS-ADRDA	National Institute of Neurological and Communicative Diseases and Stroke/Alzheimer’s Disease and Related Disorders Association
SPC	Socio-professional category
MG-CFA	Multi-Group Confirmatory Factor Analysis
IPQ-M	Illness Perceptions Questionnaire-Memory
AOS	Aging Opinion Survey
APQ	Aging Perceptions Questionnaire
FADS	Fear of Alzheimer’s Disease Scale
GGM	Gaussian Graphical Models
CS-coefficients	Correlation Stability Coefficients
MCAR	Missing Completely at Random
MICE	Multiple Imputation by Chained Equations
Time_a/c	Timeline acute/chronic
Time_s/d	Timeline stability/decline
Blame	Personal control (Blame)
Conseq	Consequences
Emo_Rep	Emotional representation
Ill_Coh	Illness coherence
Soc_Comp	Social comparison
Anxiety	Personal anxiety towards aging
Conseq_Pos	Consequences positive
Ctrl_Pos	Control positive
Gen_Fear	General fear;
Men_Det	Mental deterioration
Felt_A	Felt age
Des_A	Desired age
App_A	Apparent age
Age	Chronological age
EI	Expected Influence

## References

[1] Deary I.J., Corley J., Gow A.J., Harris S.E., Houlihan L.M., Marioni R.E., Penke L., Rafnsson S.B., Starr J.M. (2009). Age-associated cognitive decline. Br. Med. Bull..

[2] Corley J., Conte F., Harris S.E., Taylor A.M., Redmond P., Russ T.C., Deary I.J., Cox S.R. (2023). Predictors of longitudinal cognitive ageing from age 70 to 82 including APOE e4 status, early-life and lifestyle factors: The Lothian Birth Cohort 1936. Mol. Psychiatry.

[3] Ekström I., Josefsson M., Bäckman L., Laukka E.J. (2024). Predictors of cognitive aging profiles over 15 years: A longitudinal population-based study. Psychol. Aging.

[4] Ten Kate M., Dicks E., Visser P.J., Van Der Flier W.M., Teunissen C.E., Barkhof F., Scheltens P., Tijms B.M., Alzheimer’s Disease Neuroimaging Initiative (2018). Atrophy subtypes in prodromal Alzheimer’s disease are associated with cognitive decline. Brain.

[5] Hurt C.S., Burns A., Brown R.G., Barrowclough C. (2010). Perceptions of subjective memory complaint in older adults: The Illness Perception Questionnaire—Memory (IPQ-M). Int. Psychogeriatr..

[6] Shinan-Altman S., Werner P. (2019). Illness representations of dementia: A scoping review. Clin. Interv. Aging.

[7] Cosentino S., Devanand D., Gurland B. (2018). A Link between Subjective Perceptions of Memory and Physical Function: Implications for Subjective Cognitive Decline. J. Alzheimer’s Dis..

[8] García-García L., Fernandes-Pires J.A., Márquez-González M., Pedroso-Chaparro M.D.S., Benito-Rincón C., Pérez-Cardona L.M., Losada-Baltar A. (2025). Self-perceptions of Aging and Distress in Middle-aged and Older Adults. The Role of Perceived Control and Pleasant Activities. Span. J. Psychol..

[9] Levy B. (2009). Stereotype Embodiment: A Psychosocial Approach to Aging. Curr. Dir. Psychol. Sci..

[10] Barber S.J. (2020). The applied implications of age-based stereotype threat for older adults. J. Appl. Res. Mem. Cogn..

[11] Fernández-Ballesteros R., Bustillos A., Huici C. (2015). Positive Perception of Aging and Performance in a Memory Task: Compensating for Stereotype Threat?. Exp. Aging Res..

[12] Kotter-Grühn D., Hess T.M. (2012). The Impact of Age Stereotypes on Self-perceptions of Aging Across the Adult Lifespan. J. Gerontol. Ser. B.

[13] Wettstein M., Wahl H.-W., Drewelies J., Wurm S., Huxhold O., Ram N., Gerstorf D. (2023). Younger Than Ever? Subjective Age Is Becoming Younger and Remains More Stable in Middle-Age and Older Adults Today. Psychol. Sci..

[14] Hagger M.S., Orbell S. (2022). The common-sense model of illness self-regulation: A conceptual review and proposed extended model. Health Psychol. Rev..

[15] Petrie K.J., Jago L.A., Devcich D.A. (2007). The role of illness perceptions in patients with medical conditions. Curr. Opin. Psychiatry.

[16] Lin F., Gleason C.E., Heidrich S.M. (2012). Illness Representations in Older Adults with Mild Cognitive Impairment. Res. Gerontol. Nurs..

[17] Perry-Young L., Owen G., Kelly S., Owens C. (2018). How people come to recognise a problem and seek medical help for a person showing early signs of dementia: A systematic review and meta-ethnography. Dementia.

[18] Tyrrell M., Religa D., Fossum B., Hedman R., Skovdahl K., Hillerås P. (2021). Embarking on a memory assessment voice of older persons living with memory impairment. Dementia.

[19] Visser L.N.C., Fruijtier A., Kunneman M., Bouwman F.H., Schoonenboom N., Staekenborg S.S., Wind H.A., Hempenius L., De Beer M.H., Roks G. (2023). Motivations of patients and their care partners for visiting a memory clinic. A qualitative study. Patient Educ. Couns..

[20] Jiao Y.-C., Chang J., Liu C., Zhou S.-Y., Ji Y., Meng Y. (2023). Factors influencing the help-seeking behavior in patients with mild cognitive impairment: A qualitative study. BMC Health Serv. Res..

[21] Régner I., Huguet P. (2025). Age-based stereotype threat effects: From the laboratory to the clinical setting. Cortex.

[22] Barber S.J., Mather M., Gatz M. (2015). How Stereotype Threat Affects Healthy Older Adults’ Performance on Clinical Assessments of Cognitive Decline: The Key Role of Regulatory Fit. J. Gerontol. Ser. B Psychol. Sci. Soc. Sci..

[23] Haslam C., Morton T.A., Haslam S.A., Varnes L., Graham R., Gamaz L. (2012). “When the age is in, the wit is out”: Age-related self-categorization and deficit expectations reduce performance on clinical tests used in dementia assessment. Psychol. Aging.

[24] Mazerolle M., Régner I., Barber S.J., Paccalin M., Miazola A.C., Huguet P., Rigalleau F. (2017). Negative Aging Stereotypes Impair Performance on Brief Cognitive Tests Used to Screen for Predementia. J. Gerontol. Ser. B Psychol. Sci. Soc. Sci..

[25] Mazerolle M., Régner I., Rigalleau F., Huguet P. (2015). Stereotype Threat Alters the Subjective Experience of Memory. Exp. Psychol..

[26] Leventhal H., Meyer D., Nerenz D. (1980). The Common Sense Model of Illness Representations. Med. Psychol..

[27] Hagger M.S., Orbell S. (2003). A Meta-Analytic Review of the Common-Sense Model of Illness Representations. Psychol. Health.

[28] Hill N.L., Do J., Bratlee-Whitaker E., Turner J.R., Sillner A., Fishman C., Mogle J. (2024). Views of Aging and Subjective Cognition in Middle-Aged and Older Adults: A Systematic Review. Gerontology.

[29] Barker M., O’Hanlon A., McGee H.M., Hickey A., Conroy R.M. (2007). Cross-sectional validation of the Aging Perceptions Questionnaire: A multidimensional instrument for assessing self-perceptions of aging. BMC Geriatr..

[30] Westerhof G.J., Nehrkorn-Bailey A.M., Tseng H.-Y., Brothers A., Siebert J.S., Wurm S., Wahl H.-W., Diehl M. (2023). Longitudinal effects of subjective aging on health and longevity: An updated meta-analysis. Psychol. Aging.

[31] Klusmann V., Sproesser G., Wolff J.K., Renner B. (2019). Positive Self-perceptions of Aging Promote Healthy Eating Behavior Across the Life Span via Social-Cognitive Processes. J. Gerontol. Ser. B.

[32] French S.L., Floyd M., Wilkins S., Osato S. (2012). The Fear of Alzheimer’s Disease Scale: A new measure designed to assess anticipatory dementia in older adults. Int. J. Geriatr. Psychiatry.

[33] Westerhof G.J., Miche M., Brothers A.F., Barrett A.E., Diehl M., Montepare J.M., Wahl H.-W., Wurm S. (2014). The influence of subjective aging on health and longevity: A meta-analysis of longitudinal data. Psychol. Aging.

[34] Klaiber P., Pauly T. (2025). Daily Fluctuations in Subjective Age among Older Adults: Links with Stressors, Positive Events, and Emotional Reactions. Gerontology.

[35] Aftab A., Lam J.A., Thomas M.L., Daly R., Lee E.E., Jeste D.V. (2022). Subjective age and its relationships with physical, mental, and cognitive functioning: A cross-sectional study of 1,004 community-dwelling adults across the lifespan. J. Psychiatr. Res..

[36] Nelson A.P., O’Connor M.G. (2008). Mild Cognitive Impairment: A Neuropsychological Perspective. CNS Spectr..

[37] Delrieu J., Ceccaldi M., Epelbaum S., Gabelle A., Krolak-Salmon P., Paquet C., Vellas B. (2018). La maladie d’Alzheimer au stade prodromal: Comment pouvons-nous la diagnostiquer et la prendre en charge? Devons-nous le faire?. Année Gérontologique.

[38] Delage É., Rouleau I., Akzam-Ouellette M.-A., Rahayel S., Filiatrault M., Joubert S. (2025). Patterns of cortical thickness in MCI patients with and without semantic impairment. Brain Cogn..

[39] Epskamp S., Rhemtulla M., Borsboom D. (2017). Generalized Network Psychometrics: Combining Network and Latent Variable Models. Psychometrika.

[40] Hevey D. (2018). Network analysis: A brief overview and tutorial. Health Psychol. Behav. Med..

[41] Folstein M.F., Folstein S.E., McHugh P.R. (1975). “Mini-mental state”: A practical method for grading the cognitive state of patients for the clinician. J. Psychiatr. Res..

[42] Thomas-Antérion C., Ribas C., Honoré-Masson S., Berne G., Rule J.H., Laurent B. (2003). Le questionnaire de plainte cognitive (QPC): Un outil de recherche de plainte suspecte d’évoquer une maladie d’Alzheimer. Année Gérontologique.

[43] Yesavage J.A., Brink T.L., Rose T.L., Lum O., Huang V., Adey M., Leirer V.O. (1982). Development and validation of a geriatric depression screening scale: A preliminary report. J. Psychiatr. Res..

[44] Dubois B., Touchon J., Portet F., Ousset P.J., Vellas B., Michel B. (2002). «Les cinq mots» épreuve simple et sensible pour le diagnostic de la maladie d’Alzheimer. Presse Méd..

[45] Lawton M.P., Brody E.M. (1969). Assessment of older people: Self-maintaining and instrumental activities of daily living. Gerontol..

[46] Sawilowsky S.S. (2009). New Effect Size Rules of Thumb. J. Mod. Appl. Stat. Methods.

[47] Gauthier K., Morand A., Dutheil F., Alescio-Lautier B., Boucraut J., Clarys D., Eustache F., Girard N., Guedj E., Mazerolle M. (2019). Ageing stereotypes and prodromal Alzheimer’s disease (AGING): Study protocol for an ongoing randomised clinical study. BMJ Open.

[48] Besozzi A. (2017). Etude des Perceptions des Troubles Mnésiques au Cours de la Démarche Diagnostique en Consultation «Mémoire». Ph.D. Thesis.

[49] Kafer R.A., Rakowskl W., Lachman M., Hickey T. (1980). Aging Opinion Survey: A Report on Instrument Development. Int. J. Aging Hum. Dev..

[50] Tuckman J., Lorce I. (1953). Attitudes towards old people. J. Soc. Psychol..

[51] Epskamp S., Borsboom D., Fried E.I. (2018). Estimating psychological networks and their accuracy: A tutorial paper. Behav. Res. Methods.

[52] Constantin M.A., Schuurman N.K., Vermunt J.K. (2023). A general Monte Carlo method for sample size analysis in the context of network models. Psychol. Methods.

[53] Jones P.J., Ma R., McNally R.J. (2021). Bridge Centrality: A Network Approach to Understanding Comorbidity. Multivar. Behav. Res..

[54] Costantini G., Epskamp S., Borsboom D., Perugini M., Mõttus R., Waldorp L.J., Cramer A.O.J. (2015). State of the aRt personality research: A tutorial on network analysis of personality data in R. J. Res. Personal..

[55] Pons P., Latapy M. (2006). Computing communities in large networks using random walks. J. Graph Algorithms Appl..

[56] Tavakol M., Dennick R. (2011). Making sense of Cronbach’s alpha. Int. J. Med. Educ..

[57] Burger J., Isvoranu A.-M., Lunansky G., Haslbeck J.M.B., Epskamp S., Hoekstra R.H.A., Fried E.I., Borsboom D., Blanken T.F. (2023). Reporting standards for psychological network analyses in cross-sectional data. Psychol. Methods.

[58] Jamshidian M., Jalal S. (2010). Tests of Homoscedasticity, Normality, and Missing Completely at Random for Incomplete Multivariate Data. Psychometrika.

[59] Le Carret N., Lafont S., Letenneur L., Dartigues J.-F., Mayo W., Fabrigoule C. (2003). The Effect of Education on Cognitive Performances and Its Implication for the Constitution of the Cognitive Reserve. Dev. Neuropsychol..

[60] Mitchell U.A., Ailshire J.A., Brown L.L., Levine M.E., Crimmins E.M. (2016). Education and Psychosocial Functioning Among Older Adults: 4-Year Change in Sense of Control and Hopelessness. J. Gerontol. Ser. B Psychol. Sci. Soc. Sci..

[61] Bunn F., Goodman C., Sworn K., Rait G., Brayne C., Robinson L., McNeilly E., Iliffe S. (2012). Psychosocial Factors That Shape Patient and Carer Experiences of Dementia Diagnosis and Treatment: A Systematic Review of Qualitative Studies. PLoS Med..

[62] Marquet M., Boutaayamou M., Schwartz C., Locquet M., Bruyère O., Croisier J.-L., Adam S. (2018). Does negative information about aging influence older adults’ physical performance and subjective age?. Arch. Gerontol. Geriatr..

[63] Weiss D., Lang F.R. (2012). “They” are old but “I” feel younger: Age-group dissociation as a self-protective strategy in old age. Psychol. Aging.

[64] Kellermann M., Wetterauer C., Kullak-Ublick G.A., Cheetham M. (2025). The waiting room of uncertainty: Digital patient support for potentially bad news—A scoping review. Front. Digit. Health.

[65] Farina F.R., Regan J., Marquez M., An H., O’Loughlin P., Pavithra P., Taddeo M., Knight R.C., Bennett M., Lenaert B. (2023). Reducing fear and avoidance of memory loss improves mood and social engagement in community-based older adults: A randomized trial. BMC Geriatr..

[66] Fuente-Hernández L., De La Fuente-Ruiz E., Gracia-García P., Serrano-García I., Álvarez-Mon M.Á., Molina-Ruiz R.M. (2025). Using social media-based positive education to challenge negative stereotypes of aging: A quasi-experimental approach. BMC Public Health.

[67] Knight R.L., Chalabaev A., McNarry M.A., Mackintosh K.A., Hudson J. (2022). Do age stereotype-based interventions affect health-related outcomes in older adults? A systematic review and future directions. Br. J. Health Psychol..

[68] Lutfian L., Wardika I.J., Mukminin M.A., Zamroni A.H., Rizanti A.P., Chandra I.N., Widyaningtyas R., Maressa A., Maulana S. (2025). Effectiveness of health education in improving treatment adherence among patients with chronic communicable diseases: A systematic review and meta-analysis. Trop. Med. Int. Health.

[69] Miller T. (2016). A Health literacy and adherence to medical treatment in chronic and acute illness: A meta-analysis. Patient Educ. Couns..

[70] Ruth N., Tsigeman E., Likhanov M., Kovas Y., Müllensiefen D. (2023). Personality and engagement with music: Results from network modeling in three adolescent samples. Psychol. Music.

[71] Likhanov M., Fillon A., Demolliens M., ROBERT A., Darnon C., Huguet P., Regner I. (2025). Network analysis of anxiety-related traits in male and female vocational students: Identifying potential targets for educational interventions. PsyArXiv.

[72] Bulsari S., Hashim N.I., Pandya K., Kabir R. (2025). A Comparative Analysis of Dementia Strategies of Fifteen European Countries in the Context of Glasgow Declaration and WHO’s Global Action Plan. medRxiv.

[73] Hu L., Bentler P.M. (1999). Cutoff criteria for fit indexes in covariance structure analysis: Conventional criteria versus new alternatives. Struct. Equ. Model. Multidiscip. J..

[74] Chen F.F. (2007). Sensitivity of Goodness of Fit Indexes to Lack of Measurement Invariance. Struct. Equ. Model. Multidiscip. J..

[75] André S., Maulana R., Helms-Lorenz M., Telli S., Chun S., Fernández-García C.-M., De Jager T., Irnidayanti Y., Inda-Caro M., Lee O. (2020). Student Perceptions in Measuring Teaching Behavior Across Six Countries: A Multi-Group Confirmatory Factor Analysis Approach to Measurement Invariance. Front. Psychol..

[76] Marsh H.W., Hau K.-T., Wen Z. (2004). In Search of Golden Rules: Comment on Hypothesis-Testing Approaches to Setting Cutoff Values for Fit Indexes and Dangers in Overgeneralizing Hu and Bentler’s (1999) Findings. Struct. Equ. Model. Multidiscip. J..

[77] Steenkamp J.E.M., Baumgartner H. (1998). Assessing Measurement Invariance in Cross-National Consumer Research. J. Consum. Res..

