# Supplementary material for: Self-Perceptions of Aging in Older Adults: A Network Analysis of Clinical and Non-Clinical Samples
Source: Brain Sci. 2026 Feb 9;16(2):204. doi: 10.3390/brainsci16020204 (PMC12938297; doi:10.3390/brainsci16020204)
Supplement: Supplementary file 1 [file brainsci-16-00204-s001.zip › brainsci-4126951-supplementary.pdf]

Supplementary Materials

Self-perceptions of aging in older adults: A network analysis of clinical and non-clinical samples

1. Confirmatory factor analysis

A multi-group Confirmatory Factor Analysis (MG-CFA) was conducted to evaluate the factorial structure of questionnaire items and to assess the comparability of our two samples by testing measurement invariance between groups. This analysis explored whether the questionnaire operated equivalently in both groups, ensuring that any observed group differences reflected true differences in the latent constructs rather than artefacts of the measurement properties.

MG-CFA was performed on three of the four questionnaire dimensions: Self-perceptions of memory deficits (derived from the *Illness Perception Questionnaire – Memory* [5]), Attitudes towards aging (composed of items from the *Aging Opinion Survey* [49], the *Aging Perception Questionnaire* [29] and the *Fear of Alzheimer's Disease Scale* [32]), and Aging stereotypes (composed of items from *Attitudes toward old people scale* [50]).
The fourth dimension, Subjective age, consisted of three open-ended items adapted from the *Self-perceptions of aging* scale [12] Because responses were provided in a written format rather than a 5-point Likert scale as in the rest of the questionnaire, this dimension was not suitable for CFA and was therefore excluded from the analysis. In addition, the Identity factor from the Self-perceptions of memory deficits dimensions was removed from this analysis as it was assessed using dichotomous (yes/no) items.

2. Measurement invariance

Measurement invariance was measured across three distinct models. First, configural invariance tested whether the overall structure of the questionnaire (number and organization of factors) was similar between groups. Second, metric invariance examined the equality of the factor loadings, i.e., whether the latent construct was measured by the same items in our two samples. Third, scalar invariance determined the equality of item intercepts, which is a prerequisite allowing a comparison of the latent means between groups.

To validate each invariance model, model fit was assessed using four indices: 1) Root Mean Squared Error of Approximation (RMSEA) determining the discrepancy between our model and the observed data; 2) Standardised Root Mean Square Residual (SRMR) measuring the difference between observed and predicted correlations; 3) the Comparative Fit Index (CFI) comparing the complexity of the model to a simpler one; 4) Tucker-Lewis Index (TLI) which also measures the complexity of the model. All indices range from 0 to 1. Threshold values are $\leq .08$ for RMSEA and SRMR values and $\geq .90$ for CFI and TLI values [73]. Measurement invariance was evaluated using changes in fit indices with $\Delta CFI \leq 0.01$ and $\Delta RMSEA \leq 0.015$, indicating measurement invariance and supporting group comparisons. [74,75]

3. Results

3.1. Self-perceptions of memory deficits

For Self-perceptions of memory deficits, all invariance models (configural, metric and scalar invariance) demonstrated acceptable fit with RMSEA and SRMR values below .08 and CFI and TLI values exceeding .90. In addition, changes in fit indices between models met recommended threshold (ΔCFI ≤ 0.01 and ΔRMSEA ≤ 0.015) supporting full measurement invariance. These results were associated with non-significant ANOVA results indicating that latent means comparisons between groups were permissible.

Table S1. MG-CFA results for Self-perceptions of memory deficits

**Self-perceptions of memory deficits (n=213)**										
	χ^2	**df**	**CFI**	**RMSEA**	**TLI**	**SRMR**	**Model compared**	**ΔCFI**	**ΔRMSEA**	**p-value**
**Configural invariance (M1)**	411.95	320	0.943	0.052	0.925	0.062	-	-	-	-
**Metric invariance (M2)**	432.97	334	0.939	0.053	0.923	0.067	M1	0.004	0.001	0.235
**Scalar invariance (M3)**	450.22	347	0.936	0.053	0.923	0.070	M2	0.003	0.000	0.231

Note. CFI: Comparative fit index, RMSEA: root-mean-square error of approximation, TLI: Tucker-Lewis Index, SRMR: Standardized Root Mean Square Residual

3.2. Attitudes towards aging

For the Attitudes towards aging dimension, all invariance models yielded RMSEA and SRMR values close to .08 and thus within acceptable limits. However, CFI and TLI values were slightly below the recommended threshold of .90, suggesting suboptimal fit, but still interpretable given the complexity of this construct [76]. Although changes in fit models were acceptable (ΔCFI close to 0.01 and ΔRMSEA ≤ 0.015), full scalar invariance was initially not supported. Therefore, a partial scalar invariance model was established by releasing intercepts constraints on three items, identified by modification indices, while ensuring that over half of the items remained invariant and that each factor of this dimension retained several invariant items. This approach is consistent with established recommendations indicating that partial scalar invariance is sufficient for latent means comparisons [77]. The resulting model yielded acceptable fit and allowed for latent means to be compared. Nonetheless, results should be interpreted with caution.

Table S2. MG-CFA results for Attitudes towards aging

**Attitudes towards aging (n=213)**										
	χ^2	**df**	**CFI**	**RMSEA**	**TLI**	**SRMR**	**Model compared**	**ΔCFI**	**ΔRMSEA**	**p-value**
**Configural invariance (M1)**	501.80	292	0.784	0.082	0.746	0.085	-	-		-

**Metric invariance (M2)**	528.10	307	0.772	0.082	0.746	0.090	M1	0.012	0.000	0.261
**Full Scalar invariance (M3)**	557.12	322	0.757	0.083	0.742	0.093	M2	0.015	0.001	0.010
**Partial Scalar invariance (M4)**	545.45	319	0.766	0.082	0.750	0.092	M2	0.006	0.004	0.097

Note. CFI: Comparative fit index, RMSEA: root-mean-square error of approximation, TLI: Tucker-Lewis Index, SRMR: Standardized Root Mean Square Residual

3.3. Aging stereotypes

For the Aging stereotypes dimension, all invariance models showed acceptable fit with RMSEA and SRMR values ≤ .08 and CFI and TLI values close to ≥ .90. Furthermore, changes in fit indices met the recommended threshold for ΔCFI and RMSEA (respectively ≤ 0.01 and ≤ 0.015), supporting full measurement invariance. These results were associated with non-significant ANOVA results, indicating that latent means comparison was possible.

Table S3. MG-CFA results for Aging stereotypes

**Aging stereotypes (n=213)**										
	χ^2	**df**	**CFI**	**RMSEA**	**TLI**	**SRMR**	**Model compared**	**ΔCFI**	**ΔRMSEA**	**p-value**
**Configural invariance (M1)**	321.10	180	0.813	0.086	0.781	0.075	-	-	-	-
**Metric invariance (M2)**	334.00	194	0.814	0.083	0.799	0.083	M1	0.001	0.003	0.721
**Scalar invariance (M3)**	349.81	208	0.812	0.080	0.810	0.086	M2	0.002	0.002	0.326

Note. CFI: Comparative fit index, RMSEA: root-mean-square error of approximation, TLI: Tucker-Lewis Index, SRMR: Standardized Root Mean Square Residual

4. Recruitment materials

Two flyers were developed for participant recruitment: one intended for patients and one for controls.

The patient flyer was distributed by the neurologist following an initial short cognitive evaluation. It specified that eligible participants could be men or women aged over 50 years. The following information was included:
"The proposed study focuses on the "Neuropsychological Assessment and Aging" and aims to improve the conditions for assessing memory disorders.

Your participation in the study would last approximately 10 months. Participation in this research does not involve any additional financial cost to you.
We suggest that you contact one of the centers conducting this study (contact details below). During the study, you will meet with a neurologist for a standard neurological consultation, as well as a neuropsychologist for two neuropsychological assessments (memory tests, language tests, etc.) twice in nine months. Depending on the diagnosis of the first neuropsychological assessment, you may also be invited to undergo additional neuroimaging examinations."

The flyer designed for the controls was used for recruitment through public advertisement and also specified that eligible participants could be men or women aged over 50 years. The study was presented as follows: "The AGING study is a nationwide project involving five universities in France, six research laboratories, and two research centres, funded by the French National Research Agency. The aim of the study is to improve the conditions for assessing memory and its functioning.
If you are interested in participating, please do not hesitate to contact us". In addition, word-to-mouth was encouraged as an additional recruitment strategy, as indicated by the following statement: "If you know anyone who might be interested, do not hesitate to tell them about it"

5. Education-controlled ANCOVA and Network analysis

5.1. ANCOVA analyses

Means, standard deviations and *p*-values derived from one-way ANCOVAs controlling for age and education for both samples are presented in Table S4. All means, p-values and partial eta-squared values remained consistent with the results presented in the paper, with the exception of *Identity.* While previously significant when adjusting for age, the inclusion of education years as a covariate resulted in non-significant differences.

Table S4. Descriptive statistics for all variables of the questionnaire for patients and control samples

	**Patient group (hospital) *n* = 129**	**Control group (laboratory) *n* = 84**	***p*-value**	η^2 ***p***
	Mean (± SD)	Mean (± SD)		
**Self-perceptions of memory deficits**				
Identity	7.93 (±.4.45)	7.03 (±4.58)	.174	.01
Timeline acute/chronic (Time_a/c)	3.57 (±0.95)	3.65 (±0.97)	.056	.00
Timeline stability/decline (Time_s/d)	3.80 (±0.95)	3.77 (±0.98)	.828	.00
Personal control (Blame)	3.31 (±0.93)	3.63 (±0.95)	**.021**	.03

Consequences (Conseq)	2.82 (±0.90)	2.13 (±0.93)	**< .001**	.12
Emotional representation (Emo_Rep)	3.29 (±1.17)	2.47 (±1.21)	**< .001**	.10
Illness coherence (Ill_Coh)	2.74 (±1.03)	3.42 (±1.06)	**< .001**	.09
Social comparison (Soc_Comp)	2.99 (±0.93)	2.46 (±0.97)	**< .001**	.07
**Attitudes toward aging**				
Personal anxiety towards aging (Anxiety)	2.89 (±0.77)	2.72 (±0.79)	.144	.01
Consequences positive (Conseq_pos)	3.63 (±0.87)	3.53 (±0.89)	.377	.00
Control positive (Ctrl_pos)	4.36 (±0.66)	4.36 (±0.68)	.970	.00
General fear (Gen_Fear)	2.83 (±0.99)	2.53 (±1.03)	**.038**	.02
**Stereotypes of older adults**				
Mental deterioration (Men_Det)	2.42 (±0.59)	2.46 (±0.60)	.607	.00
**Subjective age**				
Felt age	62.80 (±8.39)	61.60 (±8.84)	.327	.00
Discrepancy score for Felt age (Felt_a)	-9.30 (±12.04)	-11.10 (±12.65)	.320	.00
Desired age	47.30 (±14.88)	45.60 (±15.03)	.422	.00
Discrepancy score for Desired age (Des_a)	-31.10 (±21.13)	-33.70 (±21.26)	.393	.00
Apparent age	61.90 (±5.11)	62.20 (±5.09)	.656	.00
Discrepancy score for Apparent age (App_a)	-10.18 (±7.33)	-9.58 (±7.29)	.574	.00

Note. *p*-values for means comparison were obtained with one-way ANCOVAs, controlled for age. SD = Standard Deviation

5.2. Correlation matrices

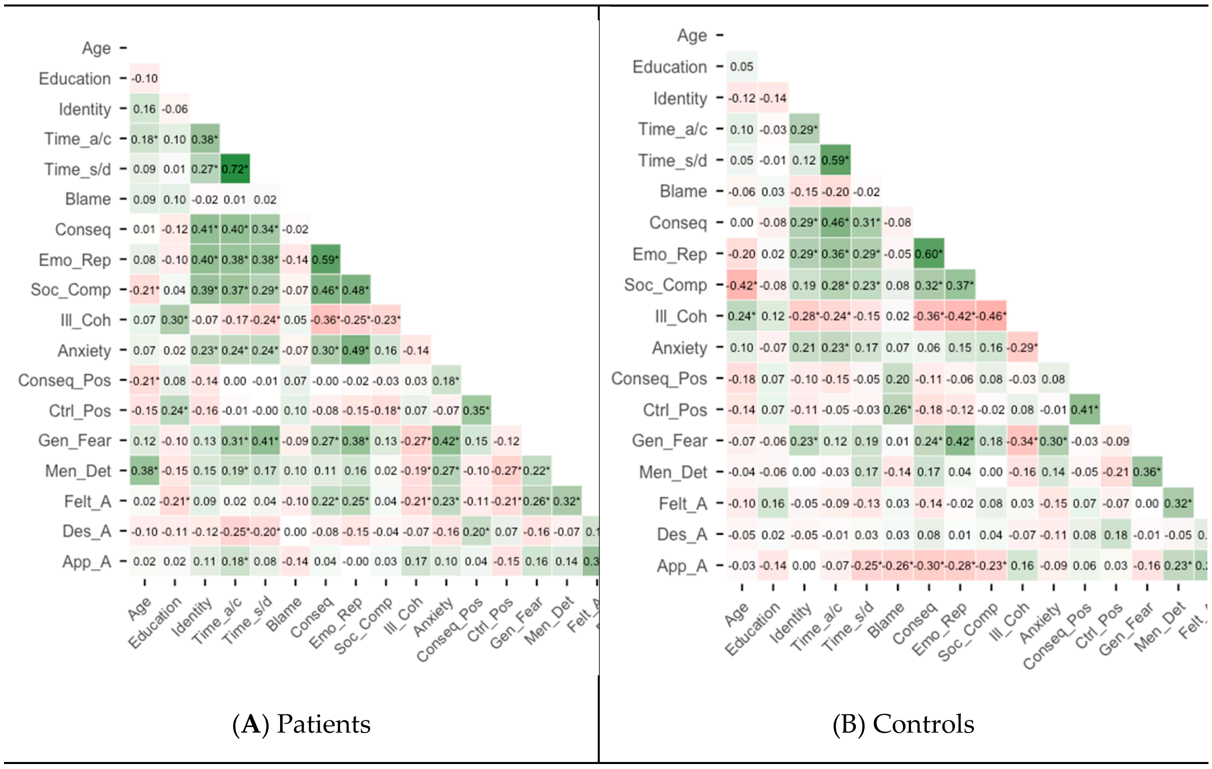

Figure S1. Correlations for all psychosocial variables for patients (Panel A) and controls (Panel B)
Note. Correlation coefficients range from –1 (in red) to 1 (in green). **p* < .05. Age : Chronological age; Education : Education years; Identity : Identity ; Time_a/c : Timeline acute/chronic; Time_s/d : Timeline stability/decline; Blame : Personal control (Blame); Conseq : Consequences; Emo_Rep : Emotional representation; Ill_Coh : Illness coherence; Soc_Comp : Social comparison; Anxiety: Personal anxiety towards aging; Conseq_Pos: Consequences positive ; Ctrl_Pos Control positive ; Gen_Fear : General fear ; Men_Det : Mental deterioration ; Felt_A : Felt age ; Des_A : Desired age ; App_A : Apparent age

The heatmap displaying the correlation matrices of the questionnaire variables is presented in Figure S1. The matrices remained consistent with the results presented in the paper, with the exception of the presence of *Education.*
In the control group, *Education* does not display significant correlations. However, in the patient sample, *Education* displays significant correlations with both *Illness Coherence* and *Control positive.*

5.3. Network analysis

Figures S2A and S2B display the network models estimated for the patient and control samples, respectively, incorporating the covariates (age and education years). These models remain largely consistent with the primary findings reported in the article, with the exception of Education and its associated edges.
In the control group, the inclusion of Education induced a shift in community detection, resulting in the emergence of a third cluster. However, the community detection for the patient group remained unchanged, suggesting that Education does not significantly alter the architecture of the representational system in the patient sample.

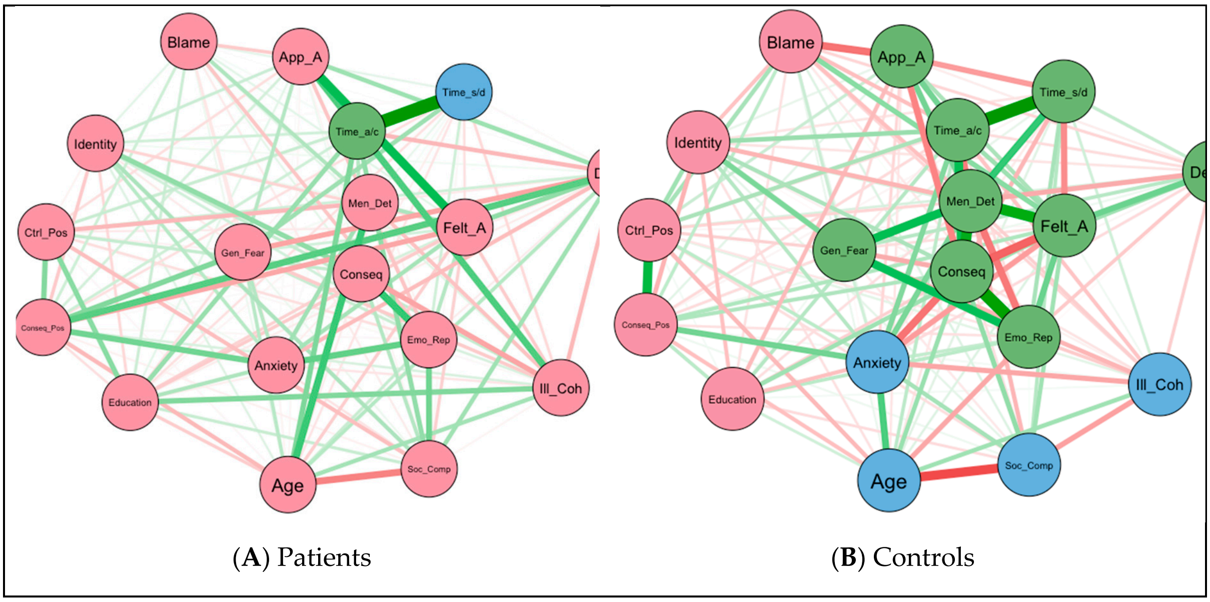

Figure S2. Network of all psychosocial variables of the AGING questionnaire in (Panel A) patients and (Panel B) controls

Note. Green lines indicate positive connections; red lines indicate negative connections; thickness of the edge represents its strength. Node colors represent the different clusters identified through community detection analyses. Age: Chronological age; Education : Education years; Identity : Identity ; Time_a/c : Timeline acute/chronic; Time_s/d: Timeline stability/decline; Blame : Personal control (Blame); Conseq: Consequences; Emo_Rep: Emotional representation; Soc_Comp: Social comparison; Ill_Coh : Illness coherence; Anxiety: Personal anxiety towards aging; Conseq_Pos: Consequences positive ; Ctrl_Pos: Control positive ; Gen_Fear : General fear; Men_Det : Mental deterioration ; Felt_A : Felt age ; Des_A : Desired age ; App_A : Apparent age

5.4. Centrality indices

Figures 3A and 3B display the centrality indices, Strength and Expected Influence, estimated for the patient and control samples, respectively, incorporating the covariates (age and education years). The centrality indices profiles remained largely consistent with the original models, as the inclusion of Education did not alter the relative importance of key variables in each group (e.g. Felt Age remained the most central node for the patients and Mental Deterioration for controls; Illness Coherence remained the node with the most negative expected influence in both networks.).

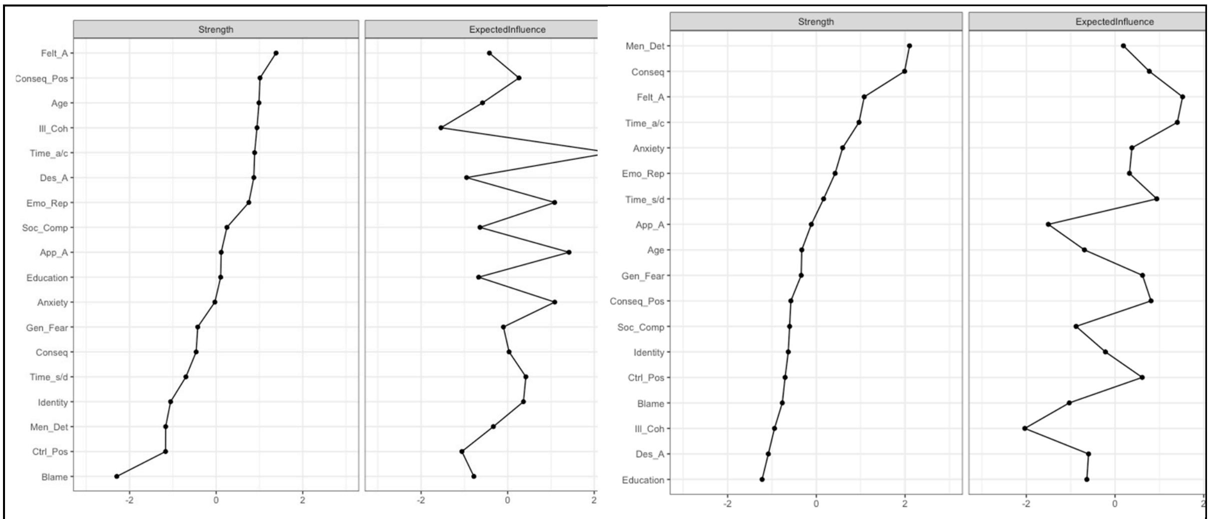

(**A**) Patients (**B**) Controls

Figure S3. Centrality indices of all psychosocial variables of the AGING questionnaire in patients (Panel A) and controls (Panel B). Strength reflects the overall degree of connectivity to each node, identifying the structural hubs of the network. Expected Influence (EI) complements this by including the sign of each edge. A positive EI indicates that a variable tends to reinforce the activity of connected nodes whereas a negative EI indicates a buffering effect on connected nodes.

Note. Age: Chronological age; Education : Education years; Identity : Identity ; Time_a/c : Timeline acute/chronic; Time_s/d: Timeline stability/decline; Blame : Personal control (Blame); Conseq: Consequences; Emo_Rep: Emotional representation; Ill_Coh : Illness coherence; Soc_Comp: Social comparison; Anxiety: Personal anxiety towards aging; Conseq_Pos: Consequences positive ; Ctrl_Pos: Control positive ; Gen_Fear : General fear; Men_Det : Mental deterioration ; Felt_A : Felt age ; Des_A : Desired age ; App_A : Apparent age
